# Brain cognition gaps reveal associations with dopamine and factors related to brain health through artificial intelligence prediction of functional connectome

**DOI:** 10.7554/eLife.104053

**Published:** 2026-09-02

**Authors:** Morteza Esmaeili, Erin Beate Bjørkeli, Robin Pedersen, Farshad Falahati, Jarkko Johansson, Kristin Nordin, Nina Karalija, Lars Bäckman, Lars Nyberg, Alireza Salami

**Affiliations:** 1 Department of Electrical Engineering and Computer Science, University of Stavanger Stavanger Norway; 2 Department of Diagnostic Imaging, Akershus University Hospital Lørenskog Norway; 3 Institute of Clinical Medicine, University of Oslo Oslo Norway; 4 Wallenberg Centre for Molecular Medicine (WCMM), Umeå University Umeå Sweden; 5 Department of Medical and Translational Biology, Umeå University Umeå Sweden; 6 Aging Research Center, Karolinska Institute and Stockholm University Solna Sweden; 7 Department of Diagnostics and Intervention, Diagnostic Radiology, Umeå University Umeå Sweden; 8 Umeå Center for Functional Brain Imaging (UFBI), Umeå University Umeå Sweden; 9 Department of Psychology, Florida State University Tallahassee United States; Amsterdam UMC Location University of Amsterdam Netherlands; Radboud University Nijmegen Netherlands

**Keywords:** cognitive resilience, aging, positron emission tomography, PET, explainable artificial intelligence, predictive modeling, deep neural networks, Human

## Abstract

A key question in human neuroscience is to understand how individual differences in brain function relate to cognitive differences. However, the optimal condition of brain function to study between-person differences in cognition remains unclear. While many studies have developed objective biomarkers to accurately predict intelligence and general cognition, consensus on domain-specific markers has not yet emerged. Brain age has been proposed as a potential candidate, but recent research suggests that brain age offers minimal additional information on cognitive decline beyond what chronological age provides, prompting a shift toward approaches focused directly on cognitive prediction. Using a deep learning approach, we evaluated the predictive power of the functional connectome during various states (resting state, movie-watching, and n-back) on episodic memory and working memory performance. Our findings show that connectomes during tasks, especially during movie-watching, predict individual differences across cognitive domains, while resting state connectomes predict episodic memory meaningfully. Furthermore, individuals with a negative brain cognition gap (where brain predictions underestimate actual performance) exhibited lower physical activity and higher cardiovascular risk compared to those with a positive gap. This shows that knowledge of the brain cognition gap provides insights into factors contributing to cognitive resilience. Further, lower PET-derived measures of dopamine binding were linked to a greater brain cognition gap, mediated by regional functional variability. Together, our findings highlight the importance of brain state in connectome-based cognitive prediction and introduce the brain cognition gap as a potentially informative, dopamine-modulated marker of vulnerability to compromise brain function.

## Introduction

Resting state functional magnetic resonance imaging (fMRI) serves as a tool to map brain function (Biswal et al., 1995; Buckner and Krienen, 2013). Functional connectivity (FC) at rest, estimated with methods to gauge correlated spontaneous activity between two or more regions, serves as a measure of functional brain integrity (Biswal et al., 1995; van den Heuvel and Hulshoff Pol, 2010). Individual differences in FC at rest are associated with differences in various cognitive domains (Ferreira and Busatto, 2013; Damoiseaux, 2017; Fox and Greicius, 2010). Associations of this nature have been reported for several large-scale networks, including the default mode network (DMN) (Andrews-Hanna et al., 2007; Salami et al., 2016; Kaboodvand et al., 2018), the frontoparietal network (Seeley et al., 2007; Avelar-Pereira et al., 2017; Avelar-Pereira et al., 2020), the dorsal attention network (Machner et al., 2021), and the salience network (Schimmelpfennig et al., 2023). Moreover, such associations have also been identified in more comprehensive investigations spanning the entire functional brain repertoire of the brain (Ferreira et al., 2016; Smith et al., 2020). Most previous reports focused on associations, not predictions, utilizing the correlation between FC and behavioral phenotypes, which tend to overfit the data and therefore fail to generalize (Shen et al., 2017). Proper cross-validation, preferably in an independent sample, is therefore important to assert reliable population-level inferences (Vul et al., 2009). Recent machine learning-based predictive frameworks offer powerful tools for assessing the predictability of individual behavioral phenotypes based on brain connectivity (Bzdok et al., 2017; Bzdok and Meyer-Lindenberg, 2018; Bzdok et al., 2018; Sui et al., 2020; Vieira et al., 2022a). In particular, deep neural network (DNN) methods have been successfully applied to behavioral and disease prediction (Plis et al., 2014; van der Burgh et al., 2017; Nguyen et al., 2018), and were initially expected to outperform other machine learning approaches (Li et al., 2018; Choi et al., 2016; He et al., 2020). However, this superiority remains debatable, as recent studies have reported comparable performance between DNNs and traditional methods (He et al., 2020; Vieira et al., 2024). Accordingly, the present study does not aim to benchmark deep learning against traditional machine learning approaches but instead uses a consistent predictive framework to examine how brain state influences the utility of FC for cognitive prediction.

The functional connectome has demonstrated predictive utility regarding trait-like cognitive phenotypes (Kong et al., 2019; Sripada et al., 2021; Chen et al., 2022; Omidvarnia et al., 2023). The predictive-modeling framework of the functional connectome has been applied to various cognitive domains, including intelligence (Jiang et al., 2021; Vieira et al., 2022b), working memory (WM) (Jangraw et al., 2018), visuospatial ability (Chen et al., 2019), attention (Rosenberg et al., 2016), creativity (Beaty et al., 2018), as well as personality traits (Hsu et al., 2018). Understanding the patterns contributing to predictions could offer insights into the functional organization underlying cognitive phenotypes, serving as biomarkers indicating current or prospective health conditions (Gabrieli et al., 2015; Finn and Todd Constable, 2016; Woo et al., 2017). Moreover, the whole-brain functional connectome acts as a fingerprint with accurate identification of subjects from a large population (Finn et al., 2015) within the same cognitive state (e.g., rest-to-rest) but also across different states (e.g., rest-to-task). Overall, past research suggests that the functional connectome is relatively robust within individuals, is unique across individuals, and can predict cognitive and personality phenotypes. However, less is known about how the predictive utility of the functional connectome depends on the brain state during which FC is measured.

Despite consensus on the value of resting state functional connectivity for mapping brain function, there is an ongoing debate about whether rest is the optimal brain state for investigating individual differences in neurocognitive function (Finn et al., 2017; Grady and Ryan, 2017; Campbell and Schacter, 2017; Geerligs and Tsvetanov, 2017). A study using data from the Human Connectome Project has shown that resting state fMRI predicts differences in brain activity during various tasks, including social, language, relational, and motor tasks (Tavor et al., 2016). This finding supports the notion that individual differences in neural activation can be predicted from resting state (Campbell and Schacter, 2017). However, results from the same dataset revealed that FC during task outperforms resting state FC in predicting individual differences in fluid intelligence, with FC during task explaining 20% of the variance compared to just 6% explained by rest (Greene et al., 2018). Consistent with this result, previous studies have investigated trait- as well as state-dependent FC, supporting the utility of an integrative approach (Avelar-Pereira et al., 2017; Geerligs and Tsvetanov, 2017). More recent studies suggested that naturalistic viewing, such as movie-watching, may serve as a happy medium between unconstrained rest and overly-constrained tasks in predicting behavior differences (Finn and Bandettini, 2021; Finn et al., 2022). Despite the presence of similar spatiotemporal activity patterns across individuals during movie-watching (Hasson et al., 2004), notable individual differences in activity and functional connectivity (Geerligs et al., 2015) persist alongside these idiosyncratic features. This suggests that tasks which align individuals’ functional connectome more closely to an optimal level, neither completely unconstrained like rest nor overly synchronized like a task, also render them easiest to identify (Finn et al., 2017). Hence, it is plausible that FC during naturalistic paradigms improves sensitivity to predict behavioral differences (Finn and Bandettini, 2021; Nordin et al., 2022).

The primary objective of this study is to determine how brain state influences the predictive utility of the functional connectome for cognitive performance, using a deep learning framework. Specifically, we test whether functional connectivity derived from different brain states differentially predicts WM and episodic memory (EM), two cores but functionally distinct cognitive domains. WM reflects the capacity to temporarily store and manipulate information and supports higher-order problem-solving, reasoning, and other key components of fluid intelligence (e.g., Cattell, 1971; Kyllonen, 2013; Unsworth et al., 2014), whereas EM entails the recollection of specific experiences and events (Tulving and Schacter, 1990), which is regarded as an important element of mind-wandering (Smallwood and Schooler, 2015) during resting state. We examine whether these domains are differentially predicted by connectomes derived from resting state, movie-watching, and n-back task fMRI.

Past studies have used neuroimaging data, including resting state, to predict brain age (Dosenbach et al., 2010; Cole and Franke, 2017; Dunås et al., 2021). These studies show that brain age, based on biological phenotypes, and their deviation from the chronological age (known as brain age gap prediction), could serve as a biomarker in characterizing disease risk (Dunås et al., 2021; Farnsworth von Cederwald et al., 2022; Jonasson et al., 2016). Importantly, an older-appearing brain has been shown to exacerbate physiological and cognitive aging and risk of mortality (Cole and Franke, 2017). A recent study demonstrated that while brain age can predict chronological age with high accuracy from MRI, its utility for predicting cognition is limited (Tetereva and Pat, 2024). Specifically, Tetereva and Pat, 2024 showed that brain age strongly tracks chronological age and that, to predict cognition, brain age largely overlapped with chronological age, such that controlling for chronological age eliminated the predictive contribution of brain age. This finding suggests that brain-age models may provide little unique explanatory power for cognitive decline beyond what is already captured by chronological age. Building on this observation and extending the concept of a brain age gap to a brain cognition gap (BCG, defined as the discrepancy between predicted and observed cognitive performance), we propose that BCG may serve as an informative marker of individual differences. If the brain predicts lower performance than is observed (i.e., a negative BCG), it may be compensating for underlying issues not yet apparent through cognitive assessments. By this view, individuals with negative BCG should be less healthy than those whose brains predict higher cognitive function than their actual performance (i.e., a positive BCG). Our second aim was to extend the concept of brain-age prediction to cognition by introducing BCG. Considering the significance of lifestyle and cardiovascular risk for maintaining healthy brain function (Nyberg et al., 2012; Karalija et al., 2019), we assess whether BCG captures individual differences beyond chronological age and examine whether individuals with positive versus negative BCG differ in lifestyle factors and cardiovascular risk, which are known contributors to brain health.

The third aim of the current study is to investigate the neurobiological underpinnings of BCG by examining the role of dopaminergic (DA) integrity. We test the hypothesis that lower DA receptor availability is associated with increased blood-oxygen-level-dependent (BOLD) signal variability, reduced functional connectome uniqueness, and larger BCG, consistent with DA’s role in modulating neural signal-to-noise ratio (SNR) and network coherence. DA is a vital neuromodulator with critical implications for motor function, reward-seeking behavior, and various higher-order cognitive functions (Bäckman et al., 2010; Bäckman et al., 2011; Volkow et al., 1998a; Juarez and Samanez-Larkin, 2019; de Boer et al., 2017; Nyberg et al., 2016; Nordin et al., 2021; Papenberg et al., 2020). Insufficient DA modulation can affect neurocognitive functions detrimentally (Bäckman et al., 2011; Nordin et al., 2021; Cools and D’Esposito, 2011; Zahrt et al., 1997; Karalija et al., 2024; Servan-Schreiber et al., 1990; Seamans and Yang, 2004; Guitart-Masip et al., 2016; Li and Rieckmann, 2014; Pedersen et al., 2023). Pharmacological studies have shown that DA depletion increases the variability of the BOLD signal, subsequently leading to less synchronized connectivity within resting state networks (Shafiei et al., 2019). Consequently, we expect individuals with inadequate DA levels to exhibit increased regional signal variability, a less unique functional connectome, and greater BCG.

Using data from the DopamiNe, Age, connectoMe, and Cognition (DyNAMiC) study (n=180, 20–79 years, 50% female) (Nordin et al., 2022), we evaluated the predictive power of the functional connectome during resting state, movie-watching, and n-back tasks for two different cognitive domains: EM and WM. Based on recent research indicating that movie-watching enhances predictability by highlighting key features of FC (Finn and Bandettini, 2021), we hypothesized that FC during movie-watching would outperform FC during rest, and possibly during task, in predicting both cognitive measures. To achieve this objective, we employed a DNN approach, a specific subtype of artificial intelligence (AI), to predict cognitive scores from the functional connectome. Deep learning approaches offer a flexible modeling framework capable of capturing complex linear and nonlinear associations in high-dimensional data (Vieira et al., 2024) and have been shown to reliably predict intelligence (Vieira et al., 2022a; Fan et al., 2020). Considering the importance of individual characteristics, such as age, in predicting behavior from FC (Omidvarnia et al., 2023), we conducted external validation of our model, initially derived from an age-heterogeneous sample, in an age-homogeneous sample (from the Cognition, Brain, and Aging (COBRA) study; Nevalainen et al., 2015). We subsequently investigated whether individuals with positive brain cognition prediction gaps differ from those with negative gaps in terms of lifestyle and cardiovascular disease (CVD) risk factors. Moreover, we tested the hypothesis of whether individuals with lower striatal dopamine D1-like receptor availability (D1DR), the brain’s most abundant DA receptor subtype, have a less distinctive FC pattern (i.e., more regional variability) and, in turn, a larger BCG. Finally, we conducted an external validation of the link between DA and the prediction gap in an independent cohort with estimates of DA D2-like receptor (D2DR) availability (Nevalainen et al., 2015).

## Results

### AI-driven predictive modeling of cognition scores from the functional connectome

We used fMRI data from the DyNAMiC project, in which each subject underwent scanning during rest, movie-watching, and WM n-back tasks. These data were parcellated into 273 nodes (264 with 9 additional subcortical nodes) using a previously published whole-brain functional atlas (Power et al., 2011). The averaged time series of 273 regions were subsequently correlated to create the FC matrix for each participant and cognitive state (rest, movie-watching, and n-back).

**Figure 1.**
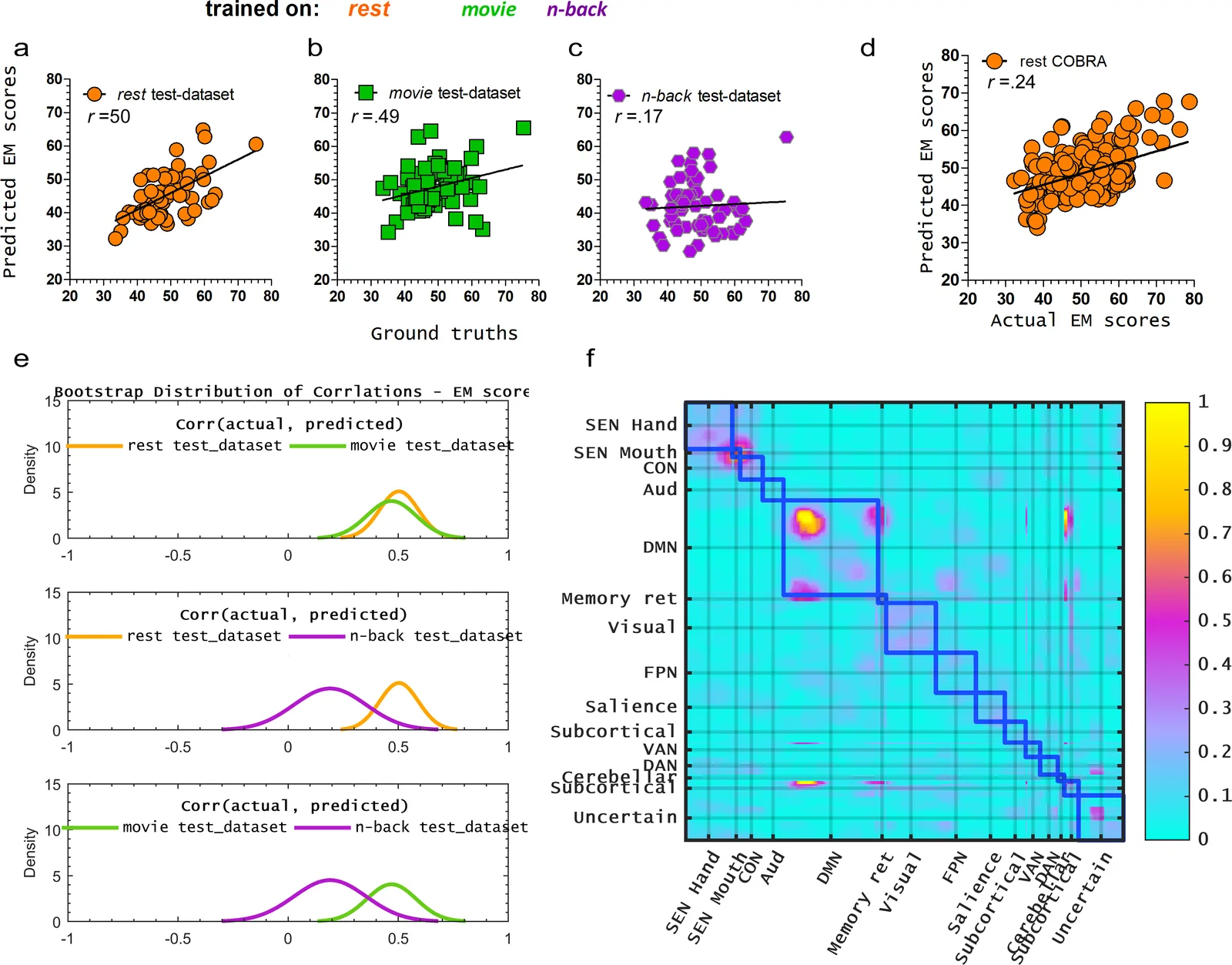
Prediction of episodic memory from resting-state and task-based functional connectivity maps. Model trained on functional connectivity maps acquired at rest predicts (**a**) episodic memory (EM) of the test dataset. The models trained on movie-watching (**b**) but not n-back (**c**) datasets predicted EM scores of the test dataset. Table 1 summarizes the p values, correlations, mean square error (MSE), and mean absolute error (MAE) for each model. Test datasets were obtained from the same cohort for rest, movie-watching, and n-back. The winning model trained on EM at rest was evaluated on the external COBRA cohort and yielded a significant prediction of EM scores (**d**). Bootstrap distributions of correlations between predicted and actual EM scores indicated no significant difference in the predictive power of EM between models trained on resting state and movie-watching data (**e**). Additionally, the bootstrap distribution revealed that models trained on resting state and movie-watching data yielded higher correlations than those trained on n-back data (**e**). Visualization of features contributing to the successful prediction of EM at rest (**f**). A Grad-CAM-derived saliency map displays the features that contributed to the model’s predictions. The hot spots overlaid on the FC map demonstrate noticeable cross-correlation contributions in ‘default mode’ (DMN) regions. Another important feature visualized by Grad-CAM includes off-diagonal hot spots reflecting inter-connections of the DMN–‘subcortical’ node.

We trained a convolutional neural network, DenselyAttention, derived from DenseNet (Huang et al., 2016), on FC matrices from each condition (resting state, movie-watching, and n-back) to generate cognition-specific prediction models of two memory domains (EM and WM). Model performance was quantified as the correlation between observed and predicted cognitive performance. Each model was then tested on all three conditions to examine the generalizability of each model across cognitive states. For example, the model trained on the resting state data (orange circles in Figure 1) was used to predict EM scores using the test dataset derived from resting state (Figure 1a), movie-watching, and n-back. Significance of our main predictions was assessed via linear correlations, and uncorrected p-values are presented in Tables 1 and 2.

**Figure 1—figure supplement 1.**
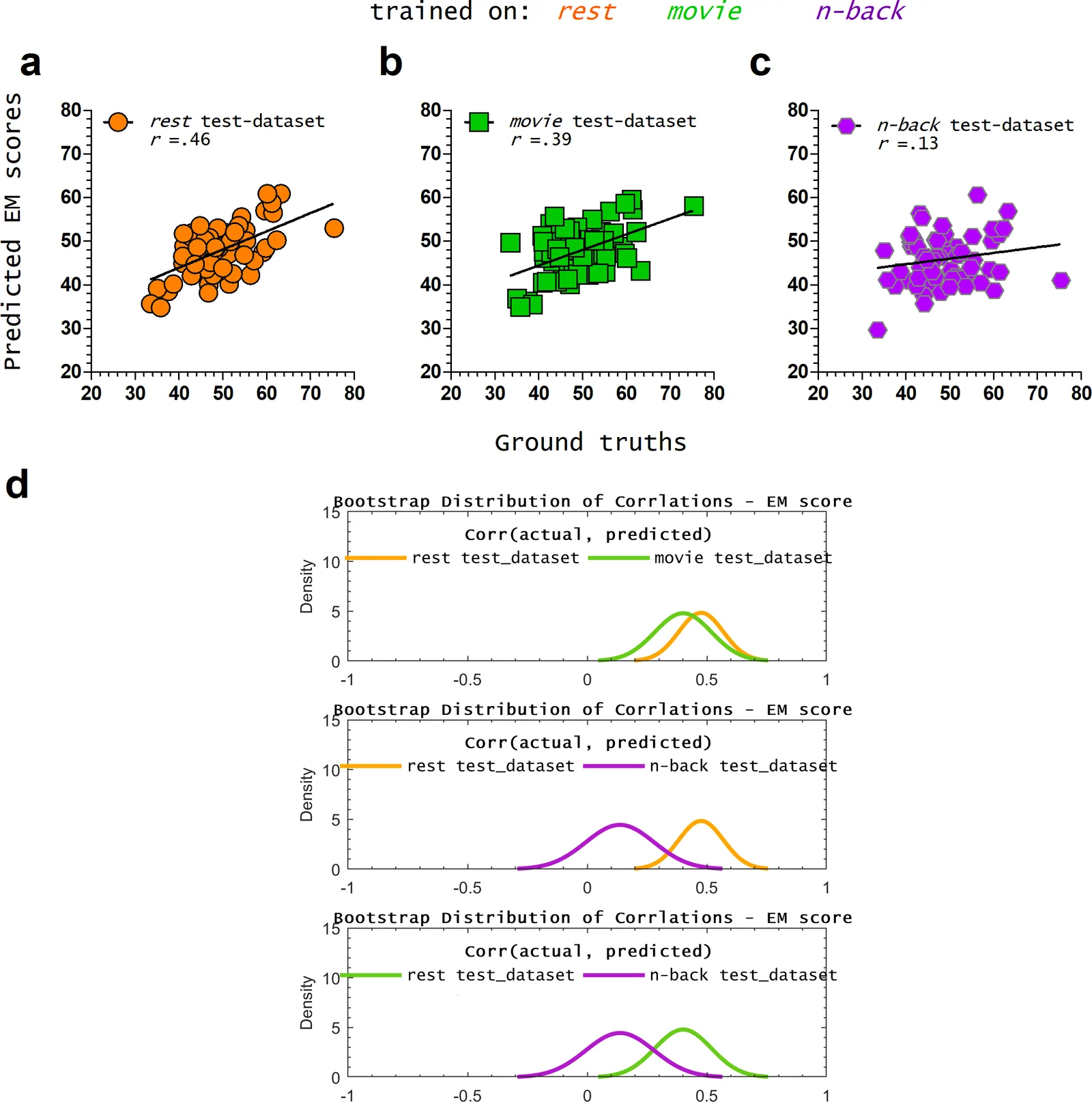
Replication of EM prediction from resting-state and task-based functional connectivity using Schaefer-300 parcellation.

**Table 1.** Correlation results for episodic memory (EM) score predictions.

Model trained on	rest			movie			n-back		
**Model tested on**	rest	movie	n-back	rest	movie	n-back	rest	movie	n-back
Correlation (*r*)	**0.50**	**0.44**	**0.38**	**0.28**	**0.49**	0.09	0.05	0.08	0.17
Correlation (*r^2^*)	**0.13**	**0.05**	**0.04**	0.03	**0.12**	0.00	–0.01	0.00	0.02
Significance	**<0.0001**	**0.0004**	**0.003**	**0.02**	**<0.0001**	0.49	0.76	0.84	0.42
MSE MAE	4.35 1.59	81.93 3.87	40.74 4.92	129.44 11.88	10.83 2.57	156.42 8.75	133.87 9.33	127.57 8.28	20.16 3.42
COBRA									
Correlation (*r*)	**0.24**								
Correlation (*r^2^*)	**0.05**								
Significance	**<0.0001**								
MSE MAE	37.62 8.28								

### Resting state and movie-watching models outperform n-back in EM prediction, with resting state offering the best generalizability

We first started with all cases in which congruent conditions were used for model building and prediction. Only models derived from the rest and movie-watching datasets yielded significant predictions of EM (Figure 1a and b and Table 1), with resting state yielding the best-performing model (*r*=0.50, p<0.0001), followed by movie-watching (*r*=0.49, p<0.0001) (Table 1). While there was no significant difference between resting state and movie-watching in predicting EM (Δ*r*=0.071, with a 95% confidence interval of [–0.097, 0.261], Figure 1e), both models yielded a markedly better EM prediction than n-back (rest vs. n-back: Δ*r*=0.333, with a 95% confidence interval of [0.054, 0.572]; movie vs. n-back: Δ*r*=0.316, with a 95% confidence interval of [0.015, 0.619], Figure 1e). Thus, the two models outperformed the n-back model (p<0.05, bootstrap test), indicating a significant improvement. To test the generalizability of these models, two types of validation analyses were performed: cross-condition and cross-dataset. In the cross-condition analysis, models trained on one condition (e.g., rest) were tested on an incongruent condition (e.g., movie-watching, n-back; Table 1). Interestingly, the model trained on resting state significantly predicted EM when tested on movie-watching (*r*=0.44, p<0.001) and n-back (*r*=0.38, p=0.003) conditions. In contrast, models trained on movie-watching or n-back could not be generalized to other conditions, were unable to significantly predict EM (p’s>0.1), except for significant generalizability from the movie to the rest condition (Table 1).

In a cross-dataset validation analysis, the best-performing model from the age-heterogeneous DyNAMiC dataset was tested on the corresponding condition in an age-homogeneous cohort from the COBRA dataset. By doing this, we found that the resting state model derived from DyNAMiC significantly predicted EM performance in the COBRA dataset (*r*=0.24, p<0.0001).

Next, we aimed to delineate the relative contributions of different brain regions for the best-performing model, the model trained on the ‘resting state data’, in predicting EM. Utilizing the Gradient-weighted Class Activation Mapping (Grad-CAM) algorithm, saliency maps were generated for the 120 FC matrices used during training of the winning model. An averaged and interpolated description of all saliency maps is depicted in Figure 1f. The saliency map highlights specific edges, especially within the default-mode network, edges between the default-mode network and subcortical areas, and edges between the default-mode and the cerebellar network. These edges, indicated by a salience intensity of ≥0.5, exert a significant influence on the model (Figure 1f).

### Movie-watching and n-back models outperform resting state in working-memory predictions, with movie-watching offering the best generalizability

We next investigated whether the superiority of resting state in predicting EM was unique to this domain, considering that previous research demonstrated the advantages of task-based fMRI and naturalistic viewing in predicting fluid intelligence (Zhao et al., 2023). To do so, we compared the predictive power of different states for WM, which is shown to be more directly associated with fluid intelligence compared to EM.

**Figure 2.**
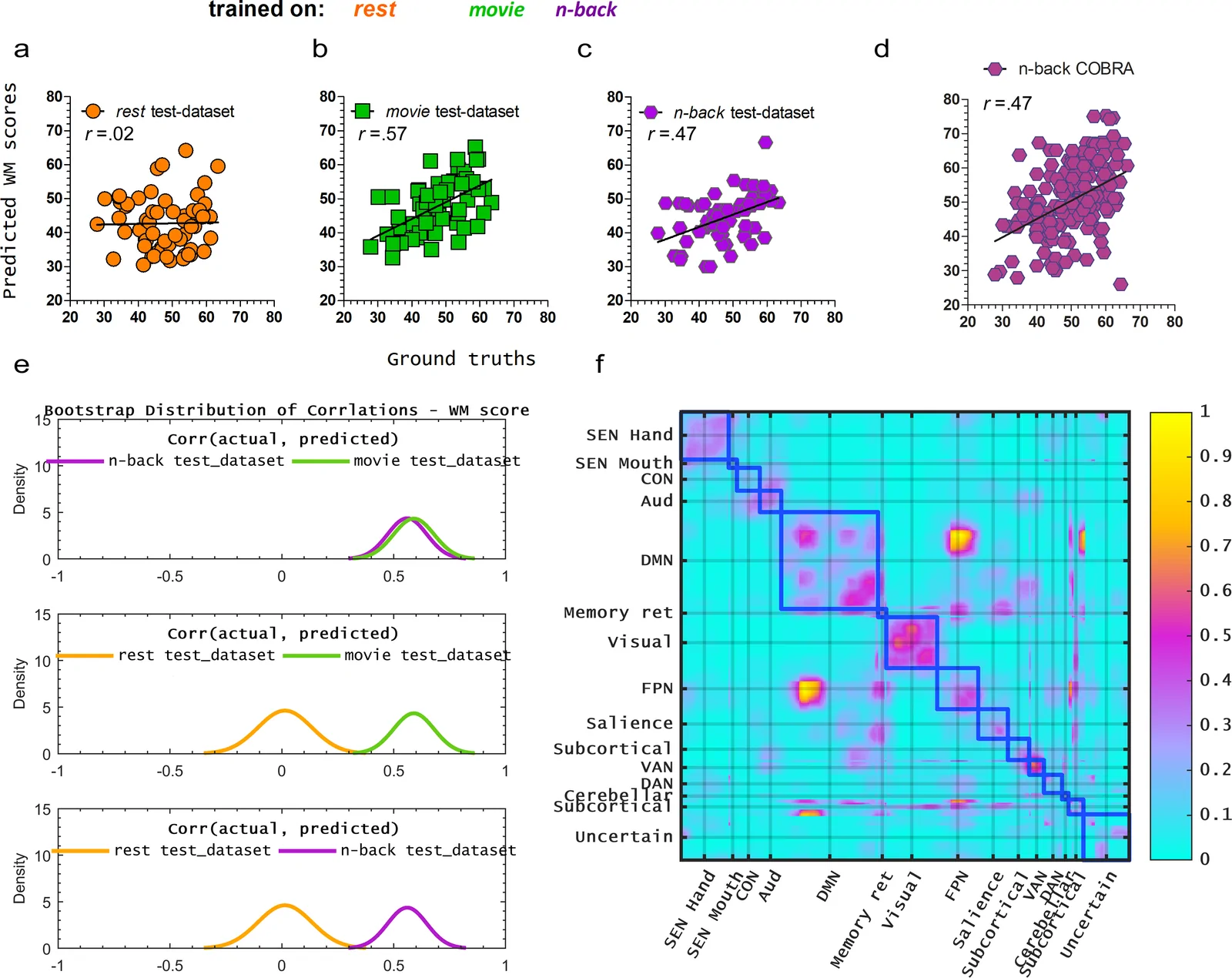
Prediction of working memory from resting-state and task-based functional connectivity maps. Model trained on FC maps acquired at rest did not significantly predict (**a**) the working memory (WM) in the test dataset. The model trained on the movie-watching dataset yielded the best-performing model in predicting WM (**b**) while the model trained on the n-back dataset (**c**) was the second-best model. Table 2 summarizes the p-values, correlation power, MSE, and MAE for each model. (**d**) Results of cross-dataset validation, where the best-performing model in the DyNAMiC dataset (i.e., movie-watching) was applied to predict WM to the COBRA dataset. However, since COBRA does not include a movie-watching paradigm, we applied the model to the n-back task in COBRA (Table 2). Bootstrap distributions of correlations between predicted and actual WM scores showed no significant difference in predictive power between models trained on movie-watching and n-back data (**e**). The bootstrap distribution revealed that models trained on movie-watching and n-back data exhibited higher correlations than those trained on resting state data (**e**). The Grad-CAM-derived saliency map highlights dominant features in the FC maps that contributed to the model’s predictions (**f**). The hot spots overlaid on an FC map demonstrate noticeable cross-correlation contributions in the ‘VAN’, ‘visual’, and to a lesser degree (<0.5) ‘DMN’. Other important features visualized by Grad-CAM include off-diagonal hot spots reflecting inter-connections of the ‘DMN’ – with ‘FPN, fronto-parietal task control’, ‘Subcortical’, and ‘Cerebellar’; ‘Cerebellar’ – ‘FPN’ node.

The model derived from the resting state failed to predict and generalize to WM (p’s>0.10; Figure 2a and Table 2). By contrast, models trained on movie-watching and n-back yielded significant predictions of WM (Figure 2b and c and Table 2), with the movie-watching model emerging as the best-performing model (*r*=0.57, p<0.0001) followed by n-back (*r*^2^=0.47, p<0.0001). While there was no significant difference between movie-watching and n-back in predicting WM (Δ*r*=0.026, with a 95% confidence interval of [0.001, 0.052], Figure 2e), these models yielded better WM prediction than resting state (Δ*r*=0.517, with a 95% confidence interval of [0.373, 0.662], Figure 2e).

**Figure 2—figure supplement 1.**
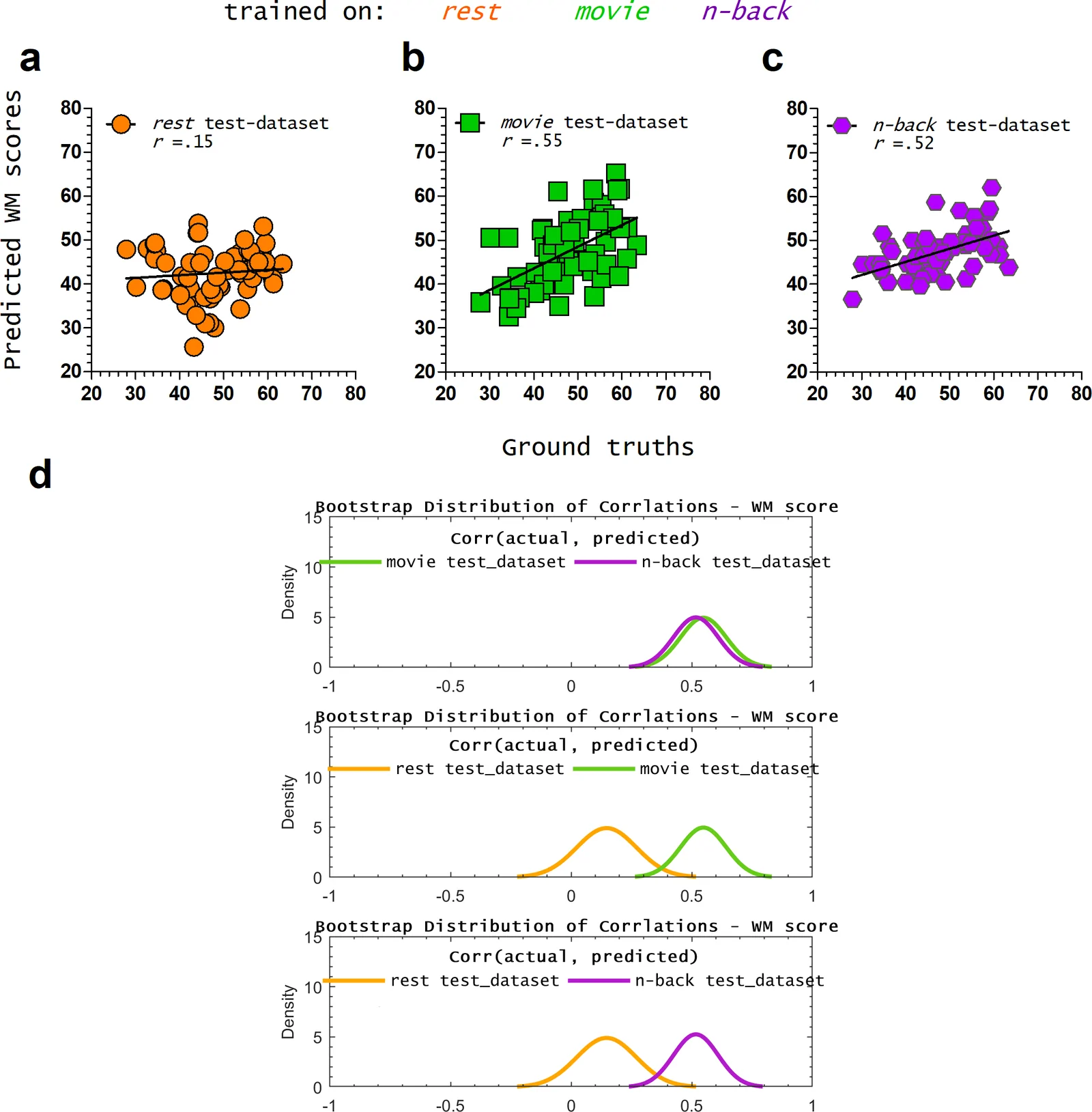
Replication of WM prediction from resting-state and task-based functional connectivity using Schaefer-300 parcellation.

**Table 2.** Correlation results for working memory (WM*)* score predictions.

Model trained on	rest			movie			n-back		
**Model tested on**	rest	movie	n-back	rest	movie	n-back	rest	movie	n-back
Correlation (*r*)	0.02	0.08	0.12	**0.42**	**0.57**	**0.46**	0.20	**0.46**	**0.47**
Correlation (*r^2^*)	–0.09	–0.05	–0.03	**0.11**	**0.17**	**0.09**	0.02	**0.05**	**0.14**
Significance MSE MAE	0.88 85.67 7.47	0.72 214.81 9.74	0.15 169.02 9.37	**<0.0001** 70.57 6.67	**<0.0001** 49.19 5.70	**0.004** 98.36 8.10	0.12 127.72 8.89	**0.0002** 116.15 8.71	**<0.0001** 61.23 6.31
COBRA									
Correlation (*r*)						**0.47**			
Correlation (*r^2^*)						**0.10**			
Significance MSE MAE						**<0.0001** 94.87 7.65			

In cross-condition validation (Table 2), the movie-watching model yielded a significant WM prediction during both resting state (*r*=0.42, p<0.0001) and n-back (*r*=0.46, p=0.004). The model derived from n-back yielded a significant WM prediction during movie-watching (*r*=0.46, p=0.0002), but not during resting state (*r*=0.20, p=0.12).

For cross-dataset validation, the best-performing model in the DyNAMiC dataset (i.e., movie-watching) was applied to predict WM in the COBRA dataset. However, since COBRA does not include a movie-watching paradigm, we applied the model to the n-back task in COBRA. This approach revealed that the model from the DyNAMiC movie-watching condition yielded a significant WM prediction in the COBRA n-back task (*r*=0.47, p<0.0001).

Overall, our results suggest that movie-watching-based WM predictions generalize across different cognitive states and datasets. This finding could be further replicated using a different functional parcellation (Figure 1—figure supplement 1, Figure 2—figure supplement 1, Supplementary file 1A and B).

The Grad-CAM algorithm generated saliency maps of 120 FC maps from the DyNAMiC dataset employed for training. Figure 2f depicts an average of all Grad-CAM generated maps. The saliency map unveils that certain edges, specifically within network connectivity of task-positive regions such as the frontoparietal task control network, dorsal/ventral attention, visual, and subcortical networks, as well as between-network connectivity. FC between task-positive dorsal and ventral attention networks, and between the DMN and the fronto-parietal network, contributed to the best-performing model derived from the movie-watching dataset. Applying two different parcellation methods (Power et al., 2011; Schaefer et al., 2018) to the DyNAMiC data indicated that parcellation resolution does not significantly impact model performance (see Figure 1—figure supplement 1, Figure 2—figure supplement 1, Supplementary file 1A and B).

### The brain cognition gap is related to physical activity levels and cardiovascular risk factors

Given the importance of lifestyle and cardiovascular health for maintaining healthy brain function (Nyberg et al., 2012; Karalija et al., 2019; Köhncke et al., 2018), we examined whether individuals with positive versus negative prediction gaps differed in physical activity habits, education, and Framingham CVD risk score. Our primary focus was on EM predictions derived from resting state data, as this was the common condition across the DyNAMiC and COBRA datasets. We computed the difference between predicted and observed EM scores to generate BCG. A positive BCG indicates that an individual’s brain predicted better-than-observed EM performance, whereas a negative BCG indicates more compromised brains relative to actual performance.

**Figure 3.**
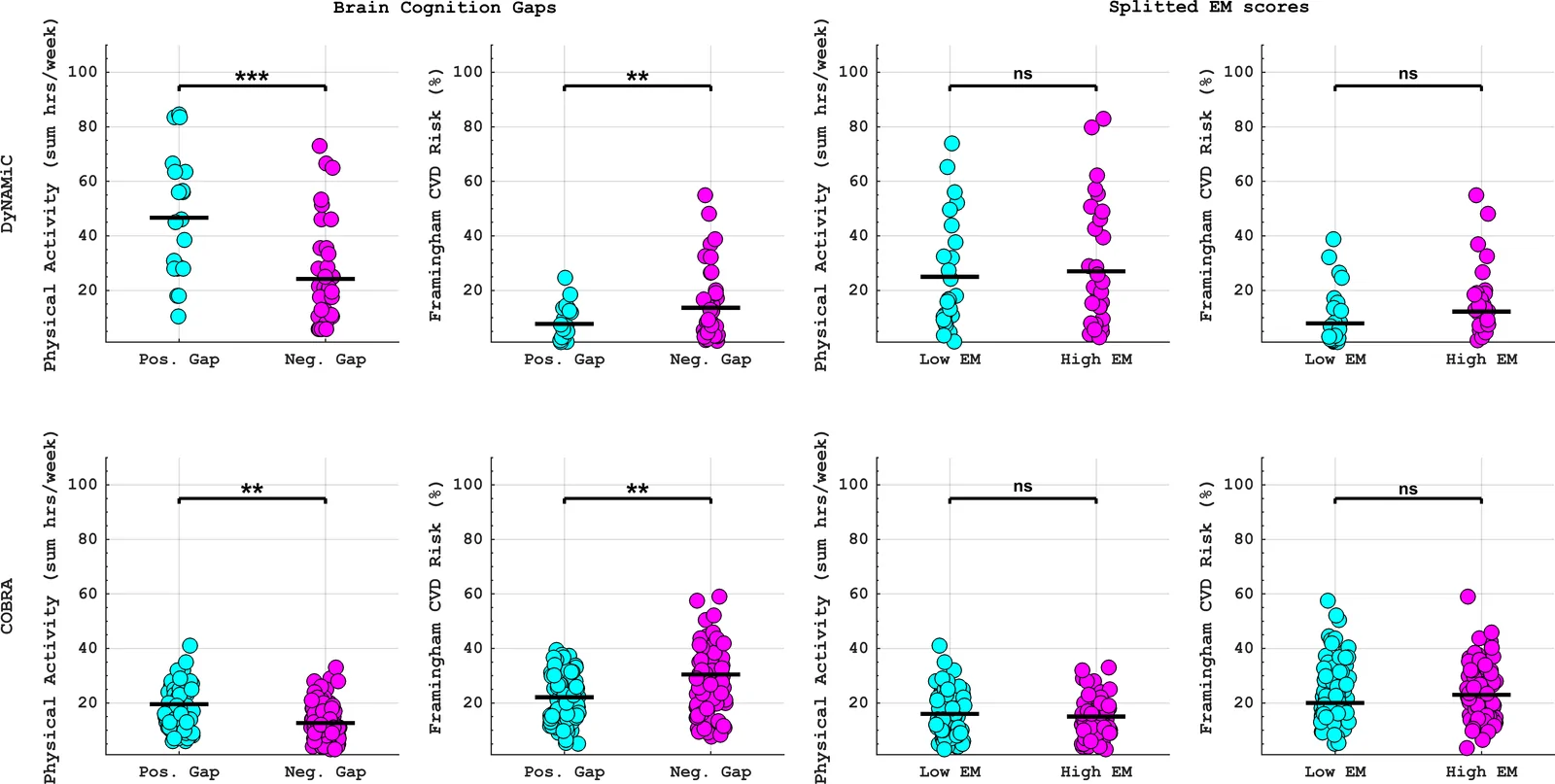
The plots compare physical activity scores (total hours per week), CVD risk, between two groups with positive and negative BAG as well as high and low memory performance in the DyNAMiC (top row) and COBRA (bottom row) datasets. *, **, and *** denote p<0.05, p<0.01, and p<0.001, respectively.

In the test sample of the DyNAMiC data (n=60) and the entire COBRA sample (n=177), we found that individuals with a negative BCG exhibited lower levels of physical activity and higher CVD risk scores compared to those with positive gaps (Figure 3). Confirmatory analysis with continuous variables revealed positive relationships between GAP and physical activity (DyNAMiC: *r* (57)=0.40, p=0.001; COBRA: *r*(166) = 0.17, p=0.03) and negative relationships between GAP and CVD risk score (DyNAMiC: *r*(57) = –0.27, p=0.03; COBRA: *r*(172) = –0.10, p=0.40). Moreover, individuals with negative BCG were less educated compared to those with positive BCG in the DyNAMiC dataset.

**Figure 3—figure supplement 1.**
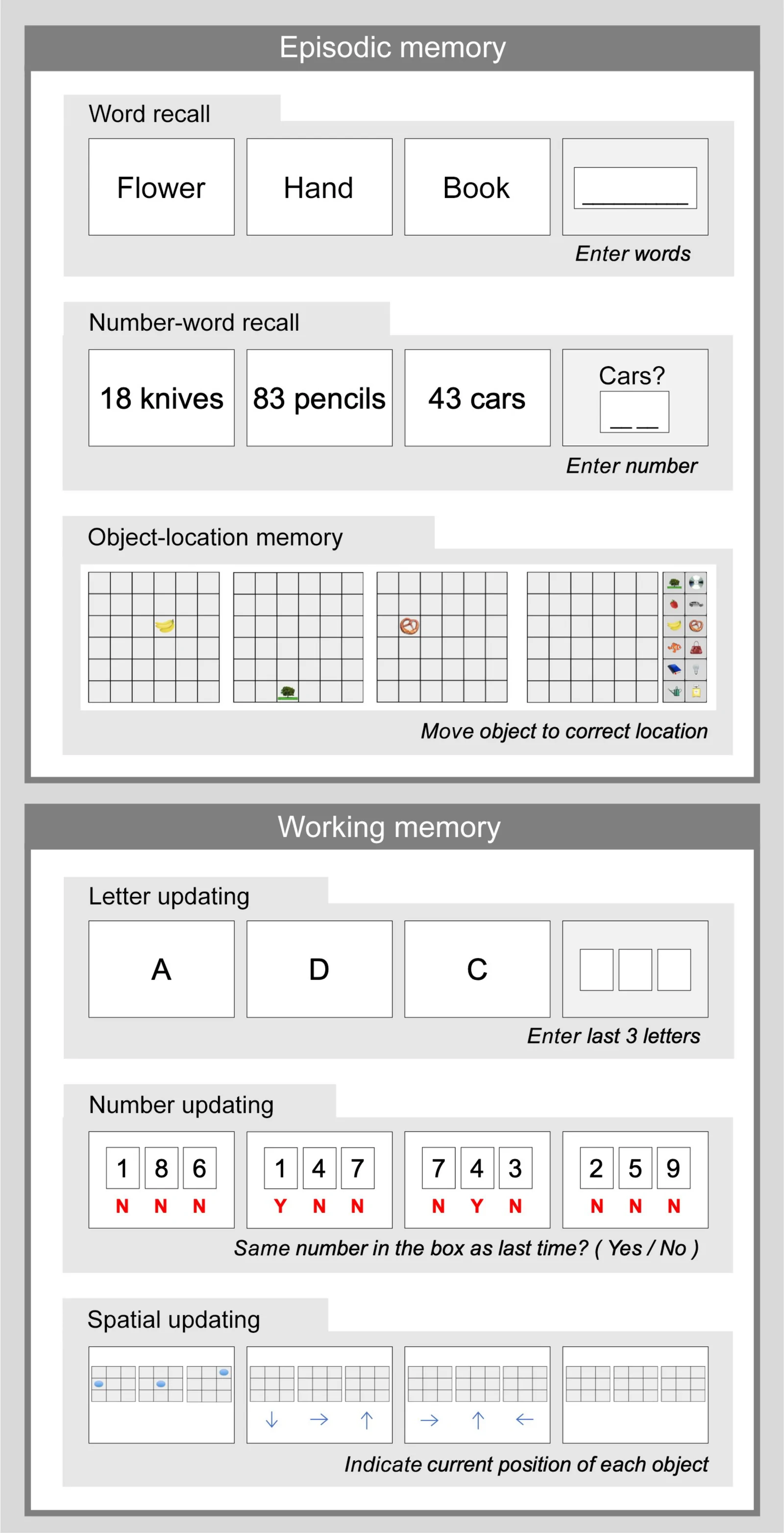
Overview of cognitive tests included in the DyNAMiC study.

To test whether cognition on its own is related to physical activity and CVD score, we conducted a median split on EM and compared physical activity and cardiovascular risk score across the two groups. In contrast to the findings related to BCG, we found no significant difference in the level of physical activity (t(58) = -0.59, p=0.56) or cardiovascular risk score (t(58) = 1.64, p=0.11) between high and low EM performers (Figure 3). These results suggest that BCG may provide additional information, beyond cognitive measures, regarding factors that contribute to cognitive resilience.

### Dopamine D1 and D2 receptor availability are associated with brain cognition gaps

Given that BCG may partly reflect variability in neural signal, one plausible neurobiological factor contributing to BCG is dopaminergic integrity. We hypothesized that inadequate DA levels might be related to increased neural SNR, thereby resulting in a less unique functional connectome, consequently leading to a greater prediction gap. We therefore initially investigated the relationship between DA receptor levels and predictive gaps across different types of DA receptors in the DyNAMiC and COBRA samples.

**Figure 4.**
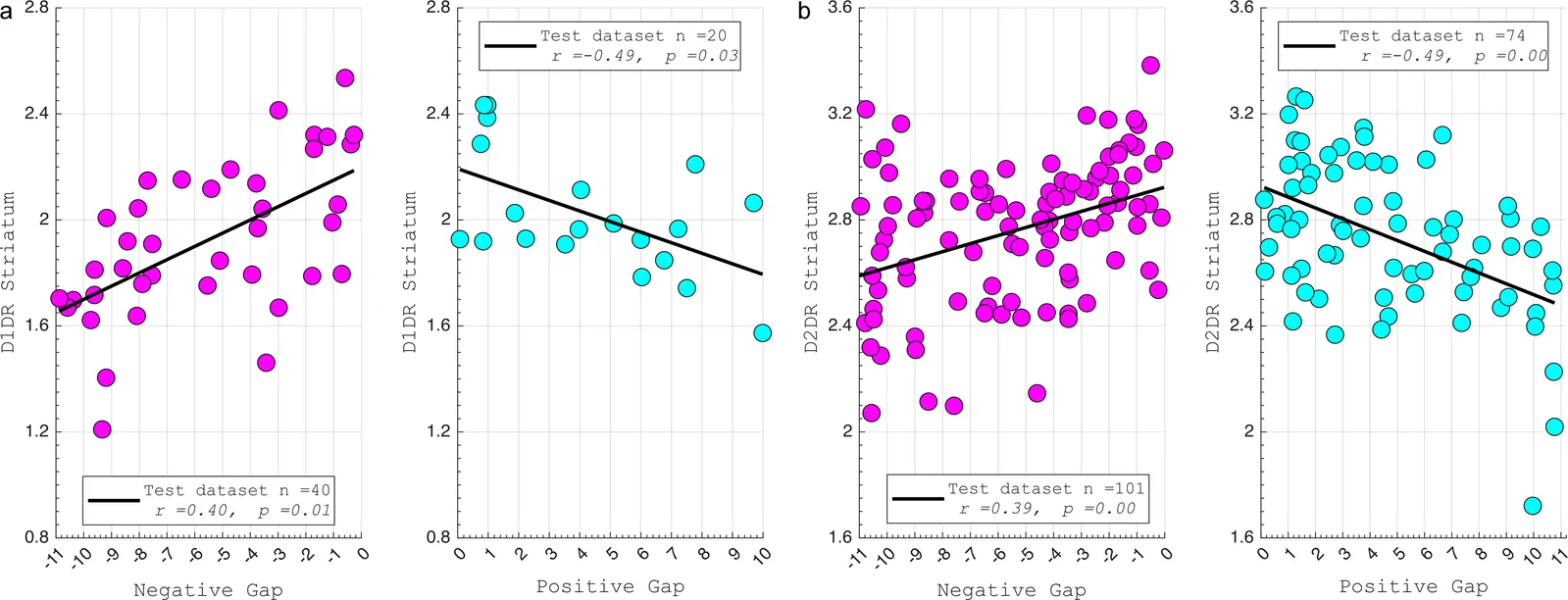
Relationship between the gap measured from predicted and actual EM scores and dopamine D1 and D2 receptors. Relationship between the gap measured from predicted and actual EM scores and dopamine D1 receptor (**a**). Negative gaps indicate that the predicted EM score was lower than actual EM scores, while the positive gaps indicate more higher predicted scores than actual EM scores. Partial correlation analysis showed a significant correlation between D1 receptor values and the measured negative and positive gaps. Relationship between the gap measured from predicted and actual EM scores and dopamine D2 receptor (**b**). Negative gaps indicate that the predicted EM score was lower than actual EM scores, while positive gaps indicate more higher predicted scores than actual EM scores. Partial correlation analysis showed a significant link of D2 receptor values to negative gaps and positive gaps.

In the DyNAMiC sample, we found a significant correlation between striatal D1DR and prediction BCG (in those with a positive gap: *r* = –0.49, p=0.03; negative gap: *r*=0.40, p=0.01) (Figure 4a), suggesting that lower D1DR is associated with greater BCG.

**Figure 4—figure supplement 1.**
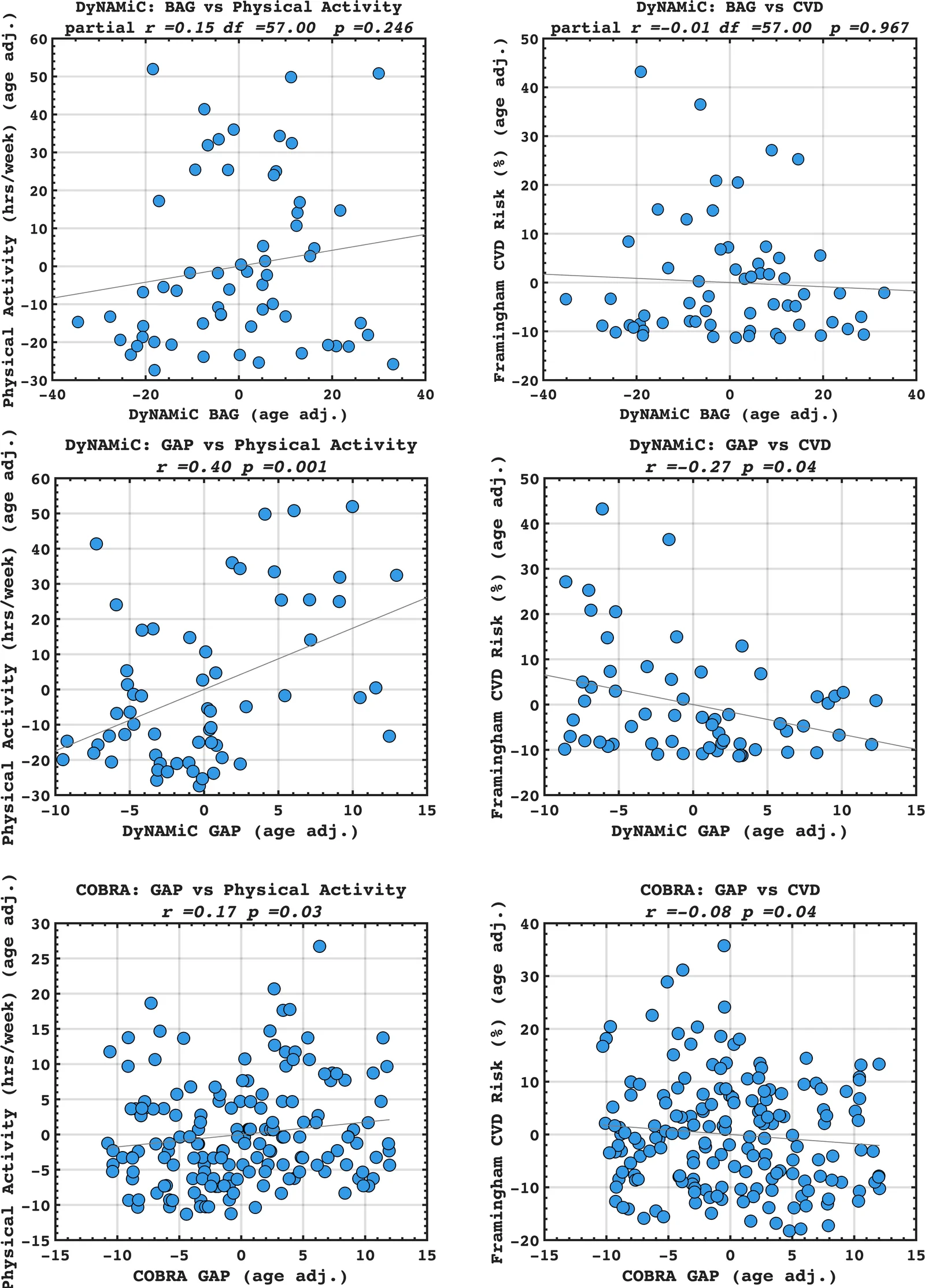
Correlations of brain age gap and brain-cognition gap with physical activity and cardiovascular disease risk.

We replicated our finding in the COBRA sample using D2-like receptor availability (D2DR), revealing a significant relationship between striatal D2DR and prediction gap (in those with a positive gap: *r* = –0.49, p=0.001; negative gap: *r*=0.39, p=0.004) (Figure 4b). Our findings provide support the view that lower D1DR/D2DR is associated with larger brain cognition prediction gaps.

Both D1DR and D2DR availability in the striatum were associated with BCG, such that lower dopamine receptor availability was linked to a greater BCG. However, these associations varied by region. For D1DR, significant correlations with BCG were observed in the caudate (positive gap: *r* = –0.37, p=0.02; negative gap: *r*=0.37, p=0.02) and putamen (positive gap: *r* = –0.53, p=0.02; negative gap: *r*=0.34, p=0.03), but not in the nucleus accumbens (positive gap: *r* = –0.25, p=0.31; negative gap: *r*=0.07, p=0.69) or the DLPFC (positive gap: *r* = –0.30, p=0.21; negative gap: *r*=0.21, p=0.21). For D2DR, both caudate (positive gap: *r* = –0.34, p=0.004; negative gap: *r*=0.36, p=0.0003) and putamen (positive gap: *r* = –0.37, p=0.002; negative gap: *r*=0.22, p=0.03) showed significant associations with BCG.

### Regional variability mediates the direct impact of dopamine on brain cognition gaps

We showed that dopamine is associated with BCG. To evaluate whether functional variability mediated this relationship, we conducted additional mediation analyses. We computed BOLD signal entropy, which estimates within-region signal variability during resting state (Shafiei et al., 2019), that was averaged across striatal regions (left caudate: MNI coordinate ≈ [–12, 12, 6], right caudate: MNI coordinate ≈ [10, 14, 2], left putamen: MNI coordinates ≈ [–24, 6, 4], right putamen: MNI coordinates ≈ [29, 1, 4]), as we expected that reduced DA may primarily impact the local functional dynamics.

In the DyNAMiC sample, within the group exhibiting a negative BCG, we observed a negative association between striatal D1DR and entropy (*r* = –0.33, p=0.04) as well as a negative association between entropy and BCG (*r* = –0.36, p=0.03). Importantly, we observed a significant indirect effect of D1DR on the gap mediated by entropy, *β*=2.41, 95% CI [0.89, 4.51], p<0.0001, explaining 56.81% of the total effect of D1DR on the gap. The direct effect of D1DR, however, was not significant, *β*=1.83, 95% CI [–0.86, 3.79], p=0.19.

Similarly, in the group with a positive BCG, entropy was negatively associated with D1DR (*r* = –0.56, p=0.01) and positively associated with BCG (*r*=0.47, p=0.04). Importantly, an indirect effect of D1DR on BCG through entropy was observed, *β* = –6.78, 95% CI [–11.26,–3.05], p<.0001, accounting for 89.41% of the D1DR effect on the gap. The direct effect of D1DR was again non-significant, *β* = –0.803, 95% CI [–0.48, 2.27], p=0.26. These findings suggest that lower D1DR levels contribute to increased signal variability, which in turn leads to reduced specificity of FC and, consequently, a larger BCG.

In the COBRA sample, within the group with a negative BCG, we observed a negative association between striatal D2DR and entropy (*r* = –0.22, p=0.03) as well as a negative association between entropy and BCG (*r* = –0.27, p=0.007). In the group with the positive BCG, entropy was negatively associated with D2DR (*r* = –0.26, p=0.03) and positively associated with BCG (*r*=0.25, p=0.03). Moreover, we detected a significant indirect effect of D2DR on both the negative and positive gap groups through entropy. For the negative gap, *β*=2.18, 95% CI [0.01, 4.25], p=0.04, accounting for 63.43% of the D2DR effect on the gap; and for the positive gap, *β* = –2.18, 95% CI [–3.87,–0.04], p=0.04, accounting for 61.49% of the D2DR effect on the gap. Similar to the results reported for D1DR, these findings suggest that lower D2DR levels contribute to increased signal variability, which in turn may lead to reduced specificity of FC and, consequently, a larger BCG.

## Discussion

Using deep learning models, we examined the predictive power of the functional connectome during various states (resting state, movie-watching, and n-back) on two different cognitive domains (EM and WM). Both rest and movie-watching states yielded significant predictions of EM, with the model derived from resting state generalized across states and datasets. Differences between the DyNAMiC and COBRA datasets make cross-dataset prediction a harder problem, as the age ranges of samples significantly vary, and prior studies highlight the importance of individual characteristics like age in predicting behavior from FC (Omidvarnia et al., 2023). In line with this, model performance decreased when predicting EM in the COBRA sample, whereas prediction of WM remained largely unchanged. Thus, validation outcomes suggest that the models, particularly those predicting WM, show robustness across datasets, whereas the reduced EM performance highlights potential data-specific influences that limit generalizability. The saliency map generated from the final layer of the deep learning model indicates that certain edges within DMN, and between DMN and the subcortical network contributed significantly to the prediction. Building on a recent finding by Kurkela and Ritchy (Kurkela and Ritchey, 2024), our finding reveals that a portion of the known-memory subnetwork within the DMN, as well as a whole-brain multivariate pattern that notably encompasses interactions of the DMN with other networks, such as the subcortical network, made a more substantial contribution to prediction. Importantly, this prediction generalizes across conditions and datasets, suggesting that features derived from resting state FC serve as a relatively stable marker of individual differences in EM, though with reduced strength in COBRA. While such generalization is partly facilitated by the similarity of functional connectivity across states, it is not a trivial outcome. For instance, the model trained on movie-watching data generalized to EM prediction during rest but failed to do so for the n-back condition, even though movie-watching and n-back connectivity patterns are themselves highly correlated. This indicates that successful generalization depends not only on shared variance across states but also on the cognitive processes most relevant to the target behavior.

Our findings are in contrast to recent work suggesting that task paradigms, in general, and movie-watching, in particular, outperform resting state data in predicting cognitive performance (Greene et al., 2018; Finn and Bandettini, 2021; Finn, 2021). While previous studies have often demonstrated a superiority of task and naturalistic viewing over resting state in predicting fluid intelligence or WM (Greene et al., 2018; Finn and Bandettini, 2021), there are fewer reports of FC predicting EM (e.g., Zhu et al., 2021; Wahlheim et al., 2022), and, to our knowledge, no study has compared rest and movie-watching. While we acknowledge that the resting state represents a complex amalgamation of cognitive, emotional, and perceptual processes (Gonzalez-Castillo et al., 2021), the good prediction power of the resting state may arise from the presence of mind wandering during rest, which is strongly related to EM (Stawarczyk and D’Argembeau, 2015; Blondé et al., 2022). EM plays a crucial role in generating mental content during mind wandering, especially episodes characterized by distinct times and locations (Smallwood and Schooler, 2015; Karapanagiotidis et al., 2017).

In contrast to the EM prediction, both n-back and movie-watching connectomes yielded significant predictions of offline WM performance. Importantly, the models derived from movie-watching and n-back outperformed the resting state in WM prediction. These differences in model accuracy when predicting the same target behaviors (i.e., WM) suggest the presence of trait–state interactions. Specifically, movies and n-back enhance individual differences in WM-relevant connections. Indeed, we found that several WM-related networks (Salami et al., 2019; Salami et al., 2018; Eriksson et al., 2015; Liang et al., 2016), including the fronto-parietal, the salience, and the dorsal/ventral attention networks, contribute to prediction. Additionally, previous research showed that movie-watching alters the propagation of activity across cortical pathways (Gilson et al., 2018), particularly within and between regions involved in audiovisual processing and attention. These alterations lead to a less segregated and more integrated network organization (Betzel et al., 2020). Similarly, the n-back task has been associated with increased integration of task-positive cortico-cortical connectivity (Liang et al., 2016; Zhang et al., 2022) and striato-cortical connectivity (Salami et al., 2018). Our findings also suggest that certain task contexts strike an optimal balance between reducing neural variability and maintaining sufficient richness to capture individual differences. Prior work shows that task states quench neural variability, leading to a more reliable and predictable neural signal (Ito et al., 2020). In this context, movie-watching may represent such a sweet spot constraining neural dynamics through shared audiovisual stimulation, while simultaneously engaging a broad range of cognitive processes that preserve individual differences. Taken together, our results confirm previous findings that movie-watching is a suitable condition for studying individual differences across various cognitive domains. Nonetheless, if a movie-watching paradigm is not feasible/available, resting state still provides a robust means of studying individual differences, particularly in self-referential domains, such as EM.

Our study used a DNN architecture that features dense connections and incorporates an attentional mechanism. While our findings demonstrate that a deep learning framework can provide reasonable predictive accuracy, it is important to note that other machine learning approaches (e.g., tree-based models) may offer comparable predictive power, as suggested by prior benchmarking work (He et al., 2020; Vieira et al., 2024). Our study explicitly compares predictive power across different cognitive states (rest, movie-watching, n-back) to identify the states that best capture individual differences across domains. The relative performance of deep learning and other non-linear approaches depends on multiple factors, including sample size, model architecture, feature representation, and domain-specific characteristics of the prediction target. In this context, deep learning was employed as a flexible framework capable of modeling high-dimensional functional connectivity patterns across cognitive states, rather than as a claim of inherent methodological superiority. Thus, our goal was not to propose a universally superior prediction model, but rather to test how brain state influences predictive utility for WM and EM using a deep learning approach.

We found a significant link between BCG, lifestyle, and risk factors for vascular disease, such that individuals with a negative BCG exhibited lower levels of physical activity and higher cardiovascular risk scores compared to those with a positive BCG. This pattern was observed in both the age-heterogeneous DyNAMiC sample and the age-homogeneous COBRA sample, although the effect sizes were smaller in COBRA. This attenuation may reflect differences in sample composition, age range, and assessment of lifestyle-related variables across cohorts. BCG could serve as a potential biomarker for identifying individuals at risk (e.g., individuals with a negative gap). Previous studies suggest that the brain age prediction gap is associated with cognitive aging (Dunås et al., 2021), some aspects of physiological aging (Cole and Franke, 2017), as well as aging in other organs (Tian et al., 2023) and even mortality in older age (Cole and Franke, 2017). However, a recent study revealed that brain age accounts for only a small portion of cognitive decline compared to chronological age (Tetereva and Pat, 2024), suggesting that cognitive prediction might be more informative. Our findings build upon this concept by extending the BCG to behavioral variables, demonstrating that the BCG could provide insights regarding physical activity status, education, and cardiovascular risk – key factors contributing to cognitive reserve (Arida and Teixeira-Machado, 2020; Klil-Drori et al., 2022; Nithianantharajah and Hannan, 2009; Cabeza et al., 2018). Note that the association with education was significant only in the DyNAMiC sample and did not reach significance in the COBRA dataset. An important caveat is that BCG can also be conceptualized as an error metric, like mean absolute error or mean square error, reflecting the extent to which models trained in one sample generalize to another. From this perspective, a larger gap may not only indicate individual differences related to resilience factors and dopaminergic function but also reduced model fit or generalizability across datasets. Thus, BCG likely reflects a combination of meaningful biological variability and methodological variance.

Critically, we found that D1DRs and D2DRs were strongly associated with the BCG, such that lower dopamine receptor levels were associated with greater gaps. More specifically, in two independent samples, we discovered greater correspondence (i.e., near zero in BCG) between brain function and cognition in individuals with higher D1DR/D2DR, whereas lower correspondence (i.e., significantly different BCG from zero) was found in individuals with lower D1DR/D2DR availability. Previous computational models proposed that DA modulates neuronal gain, which improves SNR in neural processing, contributing to more coherent activity across large-scale networks (e.g., balanced integration and segregation Pedersen et al., 2023). Past studies also showed that lower D1DR contributed to more BOLD variability in the subcortical area (Guitart-Masip et al., 2016) and less functional segregation of the striatum (Korkki et al., 2024) and the large-scale networks in aging (Pedersen et al., 2023), possibly due to increased noise (lower SNR). In support of this notion, we found for the first time that regional variability, estimated using entropy, mediated the impact of DA on BCG. Although the cross-sectional nature of our data warrants caution, this novel finding suggests that lower DA integrity relates to BOLD variability, which in turn is associated with a larger BCG.

An important caveat is that D1DR and D2DR availability do not provide a direct measure of dopamine signaling. Instead, it reflects receptor availability, which interacts with endogenous dopamine in a complex manner. PET measures of D1R and D2R availability reflect the density of unoccupied dopamine receptors and the degree to which endogenous dopamine competes with radioligand binding. D2R binding potential is sensitive to competition from synaptic dopamine, such that higher ambient dopamine generally reduces tracer binding; D1R binding, however, is less affected by endogenous dopamine under physiological conditions, reflecting more directly receptor expression levels. Previous studies demonstrated a significant association between D2R availability and dopamine synthesis capacity measured by FMT (Berry et al., 2018; Volkow et al., 1998b), suggesting that postsynaptic receptor markers may, under certain conditions, serve as a proxy for dopaminergic signaling. Developmental factors, such as the number of dopamine-producing neurons innervating the striatum, may further influence the structural and functional relationship between pre- and post-synaptic markers. By contrast, smaller studies have reported non-significant (Kienast et al., 2008; Heinz et al., 2005) or negative (Ito et al., 2011) associations, although these studies relied on [18F]FDOPA, which is considered a less precise index of dopamine synthesis than FMT. Taken together, these reports indicate that the relationship between pre- and post-synaptic markers is complex and not necessarily linear. Accordingly, our observation that lower receptor availability is associated with greater neural variability should not be interpreted as direct evidence of weaker dopaminergic signaling, but rather as reflecting the interplay between receptor density and endogenous dopamine occupancy, particularly in the case of D2DR.

Finally, we did not directly compare BCG and brain-age gap (BAG). While our focus was to investigate whether the BCG provides information about factors contributing to cognitive resilience, we acknowledge that benchmarking BCG against the brain-age gap in predicting lifestyle and vascular risk factors would be valuable. As shown in Figure 4—figure supplement 1, we did not observe significant associations between BAG and these factors, suggesting that BCG may be more sensitive to aspects of cognitive resilience. However, addressing this question lies beyond the scope of the present study, and future work should systematically compare these approaches. Our objective was not to determine whether BCG outperforms BAG, but rather to assess whether BCG provides information beyond cognitive performance itself. We also note that introducing BAG raises additional methodological questions, such as which cognitive state (rest, movie-watching, or n-back) is most appropriate for estimating biological age, warranting a dedicated investigation. Finally, we acknowledge that our main and validation samples are moderate in size for deep learning, which constrains statistical power and generalizability. Although external validation, early stopping, dropout, and regularization help mitigate overfitting, larger samples will be needed in future work to fully establish the robustness of these predictive models.

In summary, our findings reveal that while tasks like movie-watching predict both EM and WM, there are features during rest that can effectively predict internally oriented mind-wandering-type tasks, such as EM. Additionally, individuals whose brains predict poorer cognitive performance (i.e., negative gap) exhibit lower physical activity and higher cardiovascular risk compared to those whose brains predict higher cognitive function than their actual performance (i.e., positive gap). This finding suggests that our prediction model offers a potential marker to identify individuals at risk of compromised brain maintenance. Furthermore, individuals with lower DA showed less accurate cognitive prediction (larger BCG) due to increased BOLD variability and less unique and cohesive FC.

## Materials and methods

### Participants

All participants provided written informed consent, and studies were conducted in accordance with the Declaration of Helsinki and approved by the Regional Ethical Board and the local Radiation Safety Committee (reference numbers: 2012-57-31M; 2017-404-32M).

**Figure 5.**
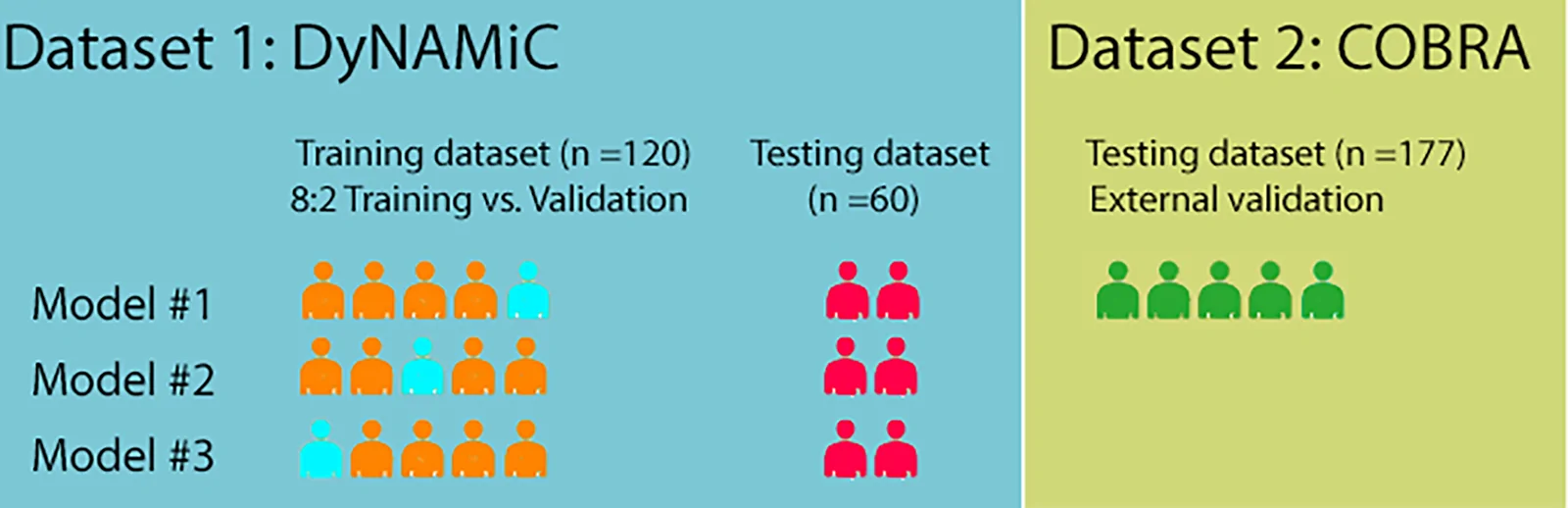
Overview of the experimental procedure and the use of datasets. We used a threefold within-sample (DyNAMiC) cross-validation where we trained our model on 120 subjects (8:2; 80% training:20% validation during training) and tested it in a separate sample of 60 subjects. The winning within-sample model was used for between-sample (COBRA) external validation.

This study used data from DyNAMiC (Nordin et al., 2022), which is a longitudinal study with a focus on changes in the brain connectome and the D1DR system. At baseline, 180 participants (20–79 years, 50% female) across the adult lifespan underwent all tests between 2017 and 2020 (Nordin et al., 2022; Figure 5). Rigorous exclusion criteria were used to recruit a sample without neurological conditions and medical treatments affecting brain functioning and cognition. Exclusion criteria included brain injury or neurological disorder, dementia, neurodevelopmental disorder, psychiatric diagnosis, psychopharmacological treatment, history of severe head trauma, substance abuse or dependence, and illicit drug use. Individuals with other chronic or severe medical conditions (e.g., cancer, diabetes, and Parkinson’s disease) were also excluded. Here, we only use data from the baseline measurement.

We used a separate sample as a testing dataset from the COBRA study (Nevalainen et al., 2015; Figure 5). COBRA is a longitudinal aging study in which 181 healthy individuals (64–68 years, 45% female) underwent baseline assessments of the brain, cognition, health, and lifestyle during 2012–2014 (Nevalainen et al., 2015). Exclusion criteria at baseline included traumatic brain injury, stroke, dementia, intellectual disability, epilepsy, psychiatric and neurological disorders, diabetes, and cancer medications, severe visual or auditory impairment, claustrophobia, and poor Swedish language skills. In the current study, we used data from 177 subjects from COBRA who underwent both MRI and PET examinations at baseline (Karalija et al., 2024; Nevalainen et al., 2015; Salami et al., 2019; Salami et al., 2018; Nyberg et al., 2023).

### Cognitive measures

The same cognitive test battery was used in DyNAMiC and COBRA (Nordin et al., 2022; Nevalainen et al., 2015; Figure 3—figure supplement 1) and assessed two cognitive domains: EM and WM. Each domain was assessed using three separate tests, including letter-, number-, and figure-based material, respectively. Participants completed all tasks on a computer and responded by either typing in words or numbers; using the computer mouse; or pressing keys marked by different colors. Each test included initial practice runs, after which testing followed across several trials. For each of the three tests within a domain, scores were summarized across trials for a measure of overall performance. A composite score of performances across the three tests was calculated and used as the measure of the cognitive domain in question (i.e., EM, WM). For each of the three tests, scores were summarized across the total number of trials. The three resulting sum scores were z-standardized and averaged to form one composite score for each domain. The standardization has been carried out independently for the training (DyNAMiC) and test (COBRA) samples.

#### Episodic memory

Tests of EM included word recall, number-word recall, and object-location recall. In word recall, participants viewed 16 Swedish concrete nouns, successively appearing on the computer screen. Each word was presented for 6 s, with an inter-stimulus interval (ISI) of 1 s. Directly following encoding, participants reported as many words as they could using the keyboard. Two trials were completed, with a combined maximum score of 32. In number-word recall, participants encoded pairs of two-digit numbers and concrete plural nouns (e.g., 46 dogs). During encoding, eight number-word pairs were presented, each for 6 s, with an ISI of 1 s. Directly after encoding, nouns were presented again, in re-arranged order, and participants had to report the two-digit number associated with each presented noun (e.g., How many dogs?). This test included two trials with a total combined maximum score of 16. The third test assessed object-location memory. Participants viewed a 6×6 square grid in which 12 objects were consecutively shown at distinct locations. Each object–position pairing was displayed for 8 s, with an ISI of 1 s. Directly following encoding, all objects were simultaneously displayed next to the grid for the participant to place in their correct position within the grid. If unable to recall the correct position of an object, participants had to guess to the best of their ability. Two trials of this test were completed, making the total combined maximum score 24.

#### Working memory

WM was examined with three tests: letter updating, number updating, and spatial updating. These tests differed from the WM *n*-back task performed during fMRI scanning. In letter-updating, capital letters (A–D) were consecutively presented on the computer screen, with participants instructed to always keep the three final letters in memory. Each letter was presented for 1 s, with an ISI of 0.5 s. When prompted, at any given moment, participants provided their responses by typing in three letters using the keyboard and provided a guessing-based answer if they failed to remember the correct letters. Four practice trials were followed by 16 test trials consisting of either 7-, 9-, 11-, or 13-letter sequences. The combined maximum number of correct answers across trials was 48 (16 trials × 3 reported letters = 48). The number-updating test followed a columnized three-back design. Three boxes were presented next to each other on the screen throughout the task, in which a single digit (Biswal et al., 1995; Buckner and Krienen, 2013; van den Heuvel and Hulshoff Pol, 2010; Ferreira and Busatto, 2013; Damoiseaux, 2017; Fox and Greicius, 2010; Andrews-Hanna et al., 2007; Salami et al., 2016; Kaboodvand et al., 2018) was sequentially presented from left to right for 1.5 s with an ISI of 0.5 s. During an ongoing sequence, participants had to judge whether the number currently presented in a specific box matched the last number presented in the same box (i.e., appearing three numbers before). For each presented number, they responded yes/no by pressing assigned keys (‘yes’=right index finger; ‘no’=left index finger). Two practice trials were followed by four test trials, each consisting of 30 numbers. The combined maximum number of correct answers across trials (after discarding responses to the first three numbers in every trial, as these were not preceded by any numbers to be matched with) was 108 (27 numbers × 4 trials). In spatial-updating, participants viewed three 3×3 square grids presented next to each other on the computer screen. At the start of each trial, a blue circular object was displayed at a random location within each grid. After 4 s, the circular objects disappeared, leaving the grids empty. An arrow then appeared below each grid, indicating that the circular object in the corresponding grid was to be mentally moved one step in the direction of the arrow. The arrows appeared stepwise from left to right in the grids, each presented for 2.5 s (ISI = 0.5 s). Prompted by three new arrows, participants mentally moved the circular objects one more time, resulting in each circular object having moved two steps from its original location at the end of each trial. Participants indicated which square the circular object in each grid had ended up in using the computer mouse. If unsure, they were instructed to guess. The test was performed across 10 test trials, preceded by five practice trials. The combined maximum number of correct location indications was 30.

### Measure of physical activity and cardiovascular disease risk

#### Physical activity

An extensive battery of self-rating questionnaires was administered in DyNAMiC and COBRA (Nordin et al., 2022; Nevalainen et al., 2015). Participants were asked to indicate the frequency (number of hours during a typical summer week; options: 1–14 h with 1 h increments, or 15+ h) and the intensity (how physically demanding on a scale from 1 = ‘not at all’ to 5 = ‘extremely’) by which they typically engage in a selection of activities relevant to life in northern Sweden. These included 15 specific activities. For the present study, we focused on physical activities and on those activities that are purely physical and that individuals are sufficiently engaged in (i.e., physically demanding ≥2.0). Each of these activities was performed by at least 20% of the participants at least once a week; e.g., walking, bicycling, jogging, strength training, household tasks, and daily work-related activities. We computed physical activity frequency (sum hrs/week) to generate physical activity scores accordingly.

#### Cardiovascular disease risk

The risk of CVD was determined via a multivariable score, according to the algorithm developed in the Framingham Heart Study (D’Agostino et al., 2008). Variables include age, sex, hypertension diagnosis, systolic blood pressure, body mass index, smoking, and diabetes mellitus. The risk estimates were derived using an algorithm proposed by D’Agostino et al., 2008, which employs Cox proportional-hazard regression models to predict the probability of developing any form of CVD within 10 years:

(1) $$\displaystyle  \hat{p}=1-S_{0}\left (t\right)exp\left (\sum \limits_{i=1}^{m}\beta _{i}\left (X_{i}-\bar {X_{i}}\right)\right)$$

with *S_0_*(*t*) being the baseline survival at follow-up *t* (*t*=10 years), *β_i_* the estimated regression coefficient, *X_i_* the log-transformed value of the *i*th risk factor, *X_i_* the corresponding mean, and *m* the number of risk factors included. Baseline survival, means, and regression coefficients were taken from the original algorithm, with DyNAMiC and COBRA participants’ risk variables inserted to compute the final scores. Risk score calculators can be found at framinghamheartstudy.org.

### Image acquisitions

Structural, functional, and neurochemical brain measures were acquired using MRI and PET at Umeå University Hospital in northern Sweden. For both DyNAMiC and COBRA, all MRI data were collected using a 3T Discovery MR750 MRI system (General Electric, Healthcare, Illinois, USA) equipped with a 32-channel phased-array head coil. PET was conducted in 3D mode with a Discovery PET/CT 690 (General Electric, WI, USA) to assess whole-brain D1DR with [^11^C]SCH23390 and D2DR with [^11^C]Raclopride at rest in DyNAMiC and COBRA, respectively. Comprehensive descriptions of MRI, PET, and cognitive testing protocols are given elsewhere (Nordin et al., 2022; Nevalainen et al., 2015). In this study, we primarily focus on those data directly pertinent to the current investigation.

#### Functional MRI

For the DyNAMiC dataset, high-resolution anatomical T1-weighted images were collected using a three-dimensional (3D) fast spoiled gradient-echo sequence with acquisition parameters of 176 sagittal slices, thickness = 1 mm, TR = 8.2 ms, TE = 3.2ms, flip angle = 12°, and a field of view (FOV)=250 × 250 mm. Whole-brain functional images were acquired during resting state, naturalistic viewing, and an n-back WM task. Functional images were acquired using a T2*-weighted single-shot echo-planar imaging (EPI) sequence, with 330 volumes collected over 12 min. The sequence provided 37 axial slices, slice thickness = 3.4 mm, 0.5 mm spacing, TR = 2000 ms, TE = 30 ms, flip angle = 80°, and FOV = 250 × 250 mm. Ten dummy scans were collected at the start of the sequence. During the resting state, participants were instructed to keep their eyes open and focus on a white fixation cross on a black background displayed on a computer screen through a tilted mirror attached to the head coil. WM was assessed in the scanner (12 min) with a numerical *n*-back task, which consisted of blocks of one-back, two-back, and three-back (Salami et al., 2019; Salami et al., 2018). During movie-watching, the participants viewed and listened to a 12-min video consisting of selected and chronologically ordered sections from the Swedish movie *Cockpit* (Johansson et al., 2023). Participants were instructed to view the movie attentively and answer a short multiple-choice questionnaire about the movie after the scanning session. We did not monitor participants’ responses to the movie, and the chosen clips were selected to be relatively neutral in emotional content. The storyline follows Valle, a recently fired pilot whose marriage has ended, as he struggles to find new employment. In a desperate attempt to secure a job at an airline specifically recruiting a female pilot, he presents himself as a woman.

In COBRA, data were collected using identical scans for resting state and n-back WM. However, the resting state scan was shorter, lasting only 6 min (Nyberg et al., 2016; Salami et al., 2018).

### Functional connectivity analysis

Functional data from all conditions (i.e., rest, movie, n-back) were preprocessed using the Statistical Parametric Mapping software package (SPM12). The functional images were first corrected for slice-timing differences and in-scanner motion, followed by registration to anatomical T1 images. Distortion correction was performed using subject-specific and T1 co-registered field maps. The functional time series were subsequently demeaned and detrended, followed by simultaneous nuisance regression and temporal high-pass filtering (threshold at 0.009 Hz) to not re-introduce nuisance signals (Hallquist et al., 2013). The nuisance regression model included mean cerebrospinal and white-matter signal and their derivatives, 24-motion parameters (Friston et al., 1996), a binary vector flagging motion-contaminated volumes exceeding framewise displacement (FD) of 0.2 mm (Power et al., 2012), in addition to an 18-parameter RETRICOR model (Glover et al., 2000; Hutton et al., 2011) of cardiac pulsation (up to third-order harmonics), respiration (up to fourth-order harmonics), and first-order cardiorespiratory interactions estimated using the PhysIO Toolbox v.5.0 (Kasper et al., 2017). Regression models for n-back included an additional set of finite impulse response (FIR) task regressors (Fair et al., 2007) to avoid false positive connectivity due to task-evoked activations (Cole et al., 2019). The FIR regression approach involved fitting mean cross-block responses for each time point within a time-locked window of equal duration to each task block (27 blocks of 20 s), extended by an additional 18 s following each block to account for the duration of the hemodynamic response function (HRF). Given that this approach linearly fits a set of binary task regressors for each time point, it is nearly identical to subtracting the mean task response, with the difference being that FIR regression is better able to handle overlapping task responses and differences in the shape of the HRF (Cole et al., 2019). The nuisance-regressed images were subsequently normalized to sample-specific group templates (DARTEL) (Ashburner, 2007) for each dataset, respectively, followed by spatial smoothing using a 6 mm FWHM Gaussian kernel to mitigate DARTEL-induced aliasing and affine-transformed to stereotactic MNI space (ICBM152NLin2009) (Fonov et al., 2011; Fonov et al., 2009). Functional images from both DyNAMiC and COBRA were preprocessed according to the steps described above, with the only exception that the RETRICOR parameters were excluded from the regression models in COBRA due to technical issues related to the respiration and cardiac traces.

#### Graph construction

For all fMRI conditions, functional time series were averaged from 273 cortical and subcortical regions, represented by 5 mm radius spheres, based on a widely employed FC parcellation (Power et al., 2011). In addition to 264 regions reported in the Power parcellation, we added nine additional regions, including some subcortical regions, such as putamen, caudate, and anterior and posterior hippocampus, identified using independent component analysis (Salami et al., 2016; Salami et al., 2022). These regions were categorized into 14 resting state networks according to a consensus partition (Power et al., 2011). To mitigate sampling from non-gray matter voxels, each parcel underwent erosion by a permissive gray matter mask (eroding voxels <.1% threshold). The averaged time series were then subjected to Pearson’s correlations, followed by Fisher’s r-to-z transformation, resulting in the creation of a 273×273 adjacency matrix for each participant, with coefficients along the main diagonals set to zero.

To further investigate the impact of network parcellation, we replicated our prediction analysis (Figure 1—figure supplement 1, Figure 2—figure supplement 1, Supplementary file 1A and B) using Schaefer parcellation (Schaefer et al., 2018), which entails 300 cortical and subcortical regions.

### Deep neural network model

Based on convolutional neural networks, deep learning is an advanced form of AI that uses multiple layers of ‘hidden’ neural networks. Deep learning methodologies are capable of automatically identifying complex patterns and representations directly from raw data using these multi-layered networks, thus eliminating the need for explicit feature engineering or manual intervention (Janiesch et al., 2021). The success of the training and learning phases depends on the model’s ability to process high-dimensional input data, extracting meaningful features from complex data. This is done while managing the number of trainable parameters, which are crucial for automated feature learning during the construction of the model.

**Figure 6.**
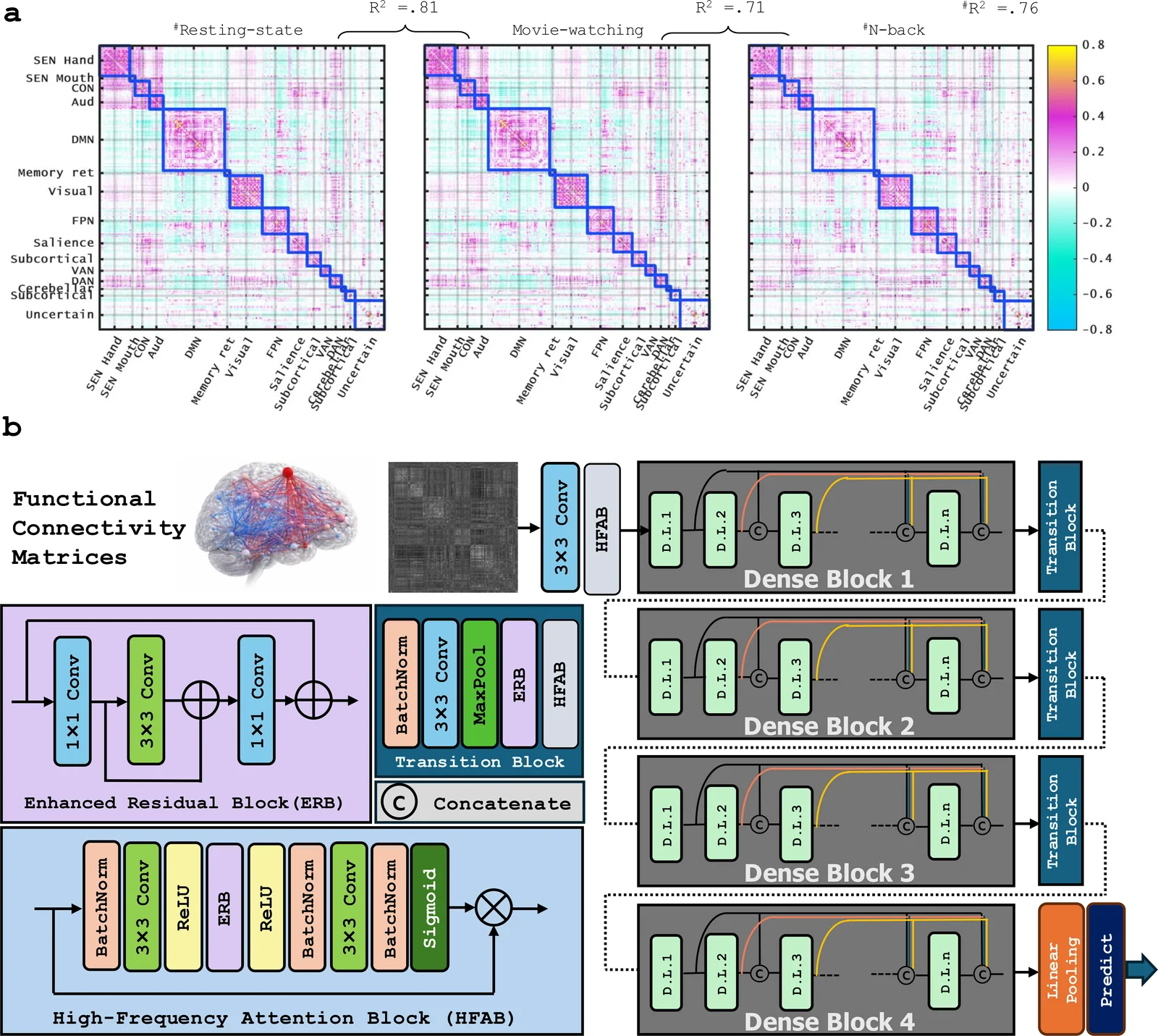
DenselyAttention network architecture for functional connectivity analysis across cognitive states. (**a**) Example of a functional connectivity map across three different cognitive states. SEN hand: SENsory hand; SEN Mouth: SENsory Mouth; CON: cingulo-operculum control network; Aud: auditory; DMN: default mode network; Memory Ret: memory retrieval network; Visual: Visual; FPN: fronto-parietal network; Salience: Salience control network; Subcortical (upper row): subcortical network included in original power parcellation; VAN: ventral attention network; DAN: dorsal attention network; Cerebellar: cerebellar network; Subcortical (lower row): additional subcortical regions, including hippocampus and caudate, added to the original power parcellation; Uncertain: regions with less known network assignment. (**b**) DenselyAttention architecture. enhanced residual block (ERB) and high-frequency attention block (HFAB) into the transition block. Note that each ‘D.L.’ layer in the table corresponds to the sequence BatchNormalization-ReLU-Conv3×3.

In this study, the inputs to our deep learning models were subject-specific FC maps with a matrix size of 273×273 (e.g., Figure 6a). We generated different versions of each FC map by replacing portions of the network. For example, we relocated the DMN network toward the last nodes, which were assigned as DAN, cerebellar, subcortical, and uncertain in Figure 6a. This approach allowed us to create a diverse set of FC matrices for each individual, each reflecting a different composition of edges and neighbors while maintaining the linear relationships exonerated in the original data. Consequently, we augmented the dataset by producing a total of 3600 FC maps from the initial set of FC maps.

Following the DenseNet framework (Huang et al., 2016), we incorporated the enhanced residual block (ERB) and high-frequency attention block (HFAB) into the dense layers (Figure 6b), termed DenselyAttention, to facilitate feature reuse in each layer. DenseNet (Huang et al., 2016) diverges from traditional methods like deepening layers or widening network structure by focusing on feature reuse and bypass settings. This results in fewer parameters than similar dense networks such as ResNet (He et al., 2015), enhances feature reuse, improves feature propagation, makes training easier, and reduces issues of gradient vanishing and model degradation.

The architecture of DenseNet is characterized by its dense connectivity pattern, which entails direct connections from each layer to all subsequent layers within its dense block (as illustrated in Figure 6b). This design ensures that every layer has access to the feature maps generated by preceding layers, thereby facilitating a seamless and efficient gradient flow throughout the network. In essence, the knowledge acquired at each layer is propagated forward, enabling the model to effectively capture intricate patterns and dependencies within the data, ultimately enhancing its ability to learn and generalize (Huang et al., 2016). Additionally, dense blocks provide a regularizing effect, reducing overfitting, particularly on tasks with smaller training datasets (Huang et al., 2016). This is suitable for this study, which includes relatively small samples. Each sequence combines operations of batch normalization (BN), rectified linear unit (ReLU), and a 3×3 convolution (Conv). Batch normalization can effectively prevent overfitting, as described by Equation 2:

(2) $$\displaystyle BN\left (x_{n}\right)=\ \alpha \left (\frac{x_{n}-\mu _{B}}{\sqrt{\sigma _{\beta }^{2}+\epsilon }}\right)+\beta,$$

where $\mu _{\beta }$, mean; $\sigma _{\beta }$, standard deviation; ϵ, random noise; α and β are adaptable variables in training. We utilized the Rectified Linear Unit (ReLU) activation function (Nair and Hinton, 2010), which activates neurons by directly outputting the input if it is positive, or zero otherwise, as outlined in Equation 2.

(3) $$\displaystyle ReLu\left (x\right)=\max \left (0,\ x\right),$$

where *x* is the input to a neuron. ReLU benefits the performance of networks with dense layers by decreasing the computation and selectively optimizing parameters.

Four dense blocks facilitate a stepwise down-sampling in the network. These blocks are connected with transition layers, which consist of a 1×1 convolutional layer followed by a 2×2 average pooling layer.

Additionally, ERB and HFAB pairs are introduced for targeted high-frequency features and residual block enhancement. The ERB-HFAB pairs are stacked sequentially at the beginning of each dense block loop (Figure 6b), with ERB and HFAB having 16 feature maps.

#### High-frequency attention block

Our approach to attentional mechanisms, specifically the HFAB, introduces a sequential attention branch inspired by edge detection. This branch rescales each position based on its neighboring pixels, efficiently focusing on high-frequency areas. The HFAB employs a 3×3 convolution to enhance both the receptive field and computational efficiency. Batch normalization is seamlessly integrated into the attention branch, introducing global statistics during inference without additional computational cost.

#### Enhanced residual block

We present ERB as an alternative to the traditional residual block. As illustrated in Figure 6b, ERB comprises a re-parameterization block (RepBlock) and a ReLU. In the training stage, the RepBlock utilizes a 1×1 convolution to either expand or contract the number of feature maps, employing a 3×3 convolution to extract features in a higher-dimensional space. Furthermore, two skip connections are integrated to mitigate training complexities. During inference, all linear transformations can be unified, facilitating the conversion of each RepBlock into a singular 3×3 convolution. Essentially, ERB capitalizes on the advantages of residual learning.

We implemented model training using TensorFlow 2.11.0 (Developers T, 2022) and Keras 2.11.0 (Chollet, 2022) as programming interfaces and trained on a fifth-generation MacBook Pro (Apple M1 MAX silicon chips, 10-core CPU, 24-core GPU, 16-core Neural Engine, 64 GB memory). For regression tasks, selecting an appropriate loss function is crucial for guiding the optimization process and ensuring accurate predictions. In this study, we opted for the mean squared error (MSE) loss function due to its suitability for regression problems. To minimize the loss function, we trained the network using the stochastic gradient descent optimizer with a learning rate of 8e^–5^ and a Nesterov momentum of 0.9 (Sutskever et al., 2013). The number of epochs was set to 100, and the batch size was set to 74. We also added dropout (Srivastava et al., 2014) after each convolutional layer, except the first one, with a rate of 0.15.

We conducted training on distinct, identical datasets extracted from DyNAMiC, each comprising FC maps generated from 120 subjects. To prepare each training dataset, we randomly shuffled data for training and validation patches, allocating 80% for training and 20% for validation. For testing, we utilized all FC maps of 60 subjects from the same sample (the age-heterogeneous DyNAMiC study), enabling us to assess model performance through threefold cross-validation (Figure 5). Each cross-validation fold was a new training with an unseen validation set for the model. Based on its performance on the testing dataset, we selected and employed the winning model for all subsequent analyses. This model, which demonstrated the best performance on the testing dataset, underwent external validation in an independent sample from the age-homogeneous COBRA study.

We initially attempted to predict both EM and WM. However, EM prediction was only reliable within and across samples for the resting state, whereas WM prediction generalized most strongly from the movie-watching condition. Because COBRA does not include a movie-watching paradigm, we could not evaluate WM prediction across datasets. For this reason, we focused on EM when examining the brain cognition.

To explore and visually represent the crucial features of the deep learning models contributing to the prediction of cognitive scores, we used the Grad-CAM technique (Selvaraju et al., 2020). This method interprets the model’s decisions by highlighting the regions of the input image with the most significant impact on the model’s output. Grad-CAM conducts a backward pass to compute the gradients of the target class score with respect to the feature maps of the final convolutional layer. We present the average heatmaps calculated for all input data to the model (Selvaraju et al., 2020). Grad-CAM saliency maps were interpreted qualitatively, with a heuristic threshold (≥0.5) applied to highlight regions with relatively higher contribution to the model’s predictions. These values do not reflect statistical significance and should therefore be interpreted descriptively. A further limitation of this study is the absence of ground truth for validating whether highlighted regions truly correspond to the features used by the model during prediction. As such, Grad-CAM provides an approximation of model attention rather than a definitive measure of feature importance. Nevertheless, Grad-CAM remains one of the most widely used and empirically validated interpretability techniques in deep learning, particularly in medical imaging applications. Its integration with established frameworks such as Keras and TensorFlow, together with its ability to generate spatial attributions that align with domain knowledge, makes it a suitable choice for the present study. Future work may incorporate complementary interpretability approaches, including adaptations of the Haufe transformation where applicable to deep learning architectures.

### Positron emission tomography (PET)

The scanning sessions started with a 5-min low-dose helical CT scan (20 mA, 120 kV, 0.8 s per revolution), obtained for attenuation correction. During scanning, a thermoplastic mask was attached to the bed surface to minimize head movement.

In DyNAMiC, a 60-min scan was performed following 350 MBq (337±27 MBq) in list-mode format. Offline re-binning of list-mode data was conducted to achieve a total of 49 frames with increasing length. In COBRA, a 55 min, 18-frame dynamic PET scan was acquired during rest following intravenous bolus injection of approximately 250 MBq 11C-raclopride (264±19 MBq). For both studies, attenuation- and decay-corrected images (47 slices, field of view = 25 cm, 256×256-pixel transaxial images, voxel size = 0.977 × 0.97×3.27 mm^3^) were reconstructed with the iterative VUE Point HD-SharpIR algorithm (GE; 6 iterations, 24 subsets, 3.0 mm post-filtering; full-width-at-half-maximum [FWHM]: 3.2 mm). The estimation of receptor availability or binding potential relative to non-displaceable binding (BP_ND_) was carried out following previously described procedures with the cerebellum as a reference region (Nordin et al., 2021). PET images were motion-corrected and co-registered with the structural T1-weighted images from the corresponding session using Statistical Parametric Mapping software (SPM12, Wellcome Department of Imaging Science, Functional Imaging Laboratory, London, UK). Motion-corrected PET data were resliced to match the spatial dimensions of MR data (1 mm^3^ isotropic, 256×256 × 256). The mean of the first five frames was used as a source for co-registration. In DyNAMiC, frame-to-frame head motion correction, with translations ranging from 0.23 to 4.22 mm (mean ± sd = 0.95±0.54 mm), revealed a trend-level difference across age-groups (age <40 and age ≥40 years), as determined by Student’s *t*-test (*t*=2.0, p=.047; mean ± sd for younger individuals = 1.07 ± 0.52, mean ± sd for older individuals = 0.90 ± 0.55). Partial-volume-effect correction was achieved using the symmetric geometric transfer matrix method for regional correction, implemented in FreeSurfer (Greve et al., 2016). An estimated point-spread-function of 2.5 mm FWHM was utilized. Regional estimates of BP_ND_ were calculated within the automated FreeSurfer segmentations employing the simplified reference tissue model (Lammertsma and Hume, 1996). In the current study, we focused on the striatal BP_ND_, calculated as an average of BP_ND_ across the caudate and putamen.

### The direct impact of dopamine on BCG through mediation analysis

To evaluate whether functional variability mediates the relationship between D1DR and prediction gap connectivity, we conducted additional mediation analyses. We first computed entropy, which estimates within-region signal variability (Shafiei et al., 2019), and then averaged this measure across all striatal regions (left caudate: MNI coordinate ≈ [–12, 12, 6], right caudate: MNI coordinate ≈ [10, 14, 2], left putamen: MNI coordinates ≈ [–24, 6, 4], right putamen: MNI coordinates ≈ [29, 1, 4]). Following the mediation analysis framework proposed by Baron and Kenny, 1986, our goal was to determine whether the association between D1/D2 receptors and BCG is mediated by regional variability (entropy) or if the indirect effect exceeds the direct association between D1/D2 receptors and BCG. To assess the statistical significance of this mediation effect, we employed the bootstrapping method as outlined by Preacher and Hayes, 2004, and age has been controlled for in all statistical analyses.

### Statistical significance analysis

Statistical analyses were carried out using SPSS (IBM Corp., V24.0.0, Armonk, NY, USA), MATLAB (The MathWorks Inc, V9.13.0 (R2022b), Natick, MA, USA), and GraphPad Prism (GraphPad Software, Inc, V5.01, CA, USA). We performed partial correlations between predicted and actual scores, as well as linear regression analyses. To investigate the relationship between generated gap variables and DA receptor availability, we controlled for age (in DyNAMiC) using partial correlation. The Mann–Whitney *U* test was used to calculate the mean differences in prediction accuracy. The level of statistical significance was set at p-value ≤0.05. For the bootstrap-based comparison of model performance (bootstrap resampling with 1000 iterations), no test statistic with an associated degree of freedom is reported. Instead, statistical inference is based on the bootstrap distribution of the difference in correlation coefficients (Δ*r*) and its 95% confidence interval. As bootstrap confidence-interval-based inference does not rely on an analytic sampling distribution, degrees of freedom are not defined for this procedure.

Out-of-sample predictive performance was quantified using the coefficient of determination (*r*^2^) computed via a sum-of-squares formulation (Poldrack et al., 2020). Unlike squared correlation coefficients, which capture only linear association, this metric evaluates how well model predictions approximate observed values relative to a baseline model. Specifically, out-of-sample *r*^2^ was defined as

$$\displaystyle  r^{2}=1-\frac{\sum \limits_{i=1}^{n}\left (y_{i}-\hat{y}_{i}\right)^{2}}{\sum \limits_{i=1}^{n}\left (y_{i}-\bar{y}\right)^{2}}$$

where *y*_i_ denotes the observed outcome in the test set, $\overset{\hat }{y}_{i}$ the corresponding model prediction, and $\bar{y}$ train denotes the mean of the outcome variable in the training set. Using the training-set mean as the baseline ensures a strictly out-of-sample evaluation and avoids information leakage. Under this formulation, positive *r*^2^ values indicate performance exceeding the null model (predicting the training mean), whereas negative values indicate worse-than-baseline performance. Because this formulation directly compares prediction error to baseline variance, it provides a more appropriate measure of predictive accuracy than correlation-based metrics, particularly in the presence of scale or offset differences between predicted and observed values (Poldrack et al., 2020).

## Data Availability

The scripts used for developing the model are available at https://github.com/MorEsm/AI-based-Prediction-of-Cognitive-Function (copy archived at Esmaeili, 2026).

## Additional files

MDAR checklist

Supplementary file 1. Replication of correlation results for EM and WM predictions using Schaefer-300 parcellation.
