# Supplementary material for: Brain cognition gaps reveal associations with dopamine and factors related to brain health through artificial intelligence prediction of functional connectome
Source: eLife. 2026 Sep 2;14:RP104053. doi: 10.7554/eLife.104053 (PMC13537765; doi:10.7554/eLife.104053)
Supplement: Supplementary file 2 [file elife-104053-supp1.docx]

Supplementary Materials for

**Brain Cognition Gaps Reveal Associations with Dopamine and Factors Related to Brain Health through Artificial Intelligence Prediction of Functional Connectome**

Esmaeili et al.

Corresponding author: Morteza Esmaeili, morteza.esmaeili@uis.no

The supplementary file includes Tables A & B

***Table A.*** *Correlation results for episodic memory (EM) score predictions – DyNAMiC dataset with Schaefer300 parcellation.*

| **Model trained on** | rest | | | movie | | | n-back | | |
| --- | --- | --- | --- | --- | --- | --- | --- | --- | --- |
| **Model tested on** | rest | movie | n-back | rest | movie | n-back | rest | movie | n-back |
| Correlation (*r*) | **0.46** | **0.38** | **0.36** | **0.34** | **0.39** | 0.09 | 0.06 | 0.15 | 0.14 |
| Correlation (*r^2^*) | **0.09** | **0.05** | **0.04** | **0.03** | **0.06** | -0.05 | -0.07 | -0.01 | -0.02 |
| Significance | **0.0002** | **0.004** | **0.005** | **0.007** | **0.002** | 0.47 | 0.67 | 0.25 | 0.28 |
| MSE | 84.18 | 112.30 | 76.82 | 129.66 | 68.23 | 138.91 | 144.22 | 95.83 | 121.45 |
| MAE | 3.66 | 4.47 | 3.37 | 5.11 | 3.02 | 5.28 | 5.40 | 3.94 | 4.96 |
|  |  |  |  |  |  |  |  |  |  |
| COBRA |  |  |  |  |  |  |  |  |  |
| Correlation | **N.A.** |  |  |  |  |  |  |  |  |
| Significance | **N.A.** |  |  |  |  |  |  |  |  |

***Table B.*** *Correlation results for working memory (WM) score predictions – DyNAMiC dataset with Schaefer300 parcellation.*

| **Model trained on** | rest | | | movie | | | n-back | | |
| --- | --- | --- | --- | --- | --- | --- | --- | --- | --- |
| **Model tested on** | rest | movie | n-back | rest | movie | n-back | rest | movie | n-back |
| Correlation (*r*) | 0.16 | 0.12 | 0.10 | **0.33** | **0.55** | **0.43** | **0.38** | **0.46** | **0.52** |
| Correlation (*r^2^*) | -0.01 | -0.03 | -0.04 | **0.03** | **0.16** | **0.09** | **0.05** | **0.10** | **0.14** |
| Significance | 0.27 | 0.36 | 0.43 | **0.01** | **<0.0001** | **0.006** | **0.003** | **<0.0001** | **<0.0001** |
| MSE | 90.90 | 92.76 | 94.61 | 87.33 | 76.51 | 82.97 | 86.45 | 81.04 | 77.43 |
| MAE | 3.22 | 3.77 | 3.89 | 3.74 | 3.12 | 3.62 | 3.79 | 3.88 | 3.29 |
|  |  |  |  |  |  |  |  |  |  |
| COBRA |  |  |  |  |  |  |  |  |  |
| Correlation |  |  |  |  |  | **N.A.** |  |  |  |
| Significance |  |  |  |  |  | **N.A.** |  |  |  |
