# Supplementary material for: Brain cognition gaps reveal associations with dopamine and factors related to brain health through artificial intelligence prediction of functional connectome
Source: eLife. 2026 Sep 2;14:RP104053. doi: 10.7554/eLife.104053 (PMC13537765; doi:10.7554/eLife.104053)
Supplement: Peer review history

# Editor report: eLife Assessment

**DOI:** 10.7554/eLife.104053.4.sa0

**Authors:** Guido van Wingen

**Affiliations:** Amsterdam UMC Location University of Amsterdam Netherlands

**Keywords:** Important, Solid

This multimodal neuroimaging study leverages fMRI, PET, and deep learning to predict memory performance. The authors introduce the brain cognition gap to link these different imaging modalities to cognition and evaluate their results in two independent cohorts. The results are **solid** and provide an **important** contribution to the literature and will be of interest to neuroscientists working at the interface of cognition, neuroimaging.

# Referee report: Reviewer #1 (Public review)

**DOI:** 10.7554/eLife.104053.4.sa1

[Editor's Note: this version has been assessed by the Reviewing Editor without further input from the original reviewers. The authors have responded to the comments raised in the previous round of review.]

Summary:

The authors attempted to identify if a new deep learning model could be applied to both resting and task state fMRI data to predict cognition and dopaminergic signaling. They found that resting state and moving watching conditions best predict episodic memory, but only movie watching predicts both episodic and working memory. A negative 'brain gap' (where the model trained on brain connectivity predicts worse performance than what is actually observed) was associated with less physical activity, poorer cardiovascular function, and lower D1R availability.

Strengths:

The paper should be of broad interest to the journal's readership, with implications for cognitive neuroscience, psychiatry, and psychology fields. The paper is very well-written and clear. The authors use two independent datasets to validate their findings, including two of the largest databases of dopamine receptor availability to link brain functional connectivity/activity with neurochemical signaling.

Comments on previous version:

I thank the authors for their extensive efforts to revise the manuscript. I have no further concerns.

# Referee report: Reviewer #2 (Public review)

**DOI:** 10.7554/eLife.104053.4.sa2

Summary:

The authors developed a deep learning model based on a DenseNet CNN architecture to predict two cognitive functions: working memory and episodic memory, from functional connectivity matrices. These matrices were recorded under three conditions: during rest, a working memory task, and a movie, and were treated as images for the CNN algorithm. They tested their model's performance across different conditions and a separate dataset with a different age distribution (using the same MRI scanner, scanning configurations, and cognitive tests). They also calculated the "brain cognition gap" based on the model trained on resting functional connectivity to predict working memory. Extending from the commonly used index "brain age," the brain cognition gap was defined as the difference between the working memory score predicted by their model (predicted working memory) and the working memory score based on the working memory test itself (observed working memory). This brain cognition gap was found to be associated with physical activity, education, and cardiovascular risk. The authors also conducted additional mediation tests to examine whether regional functional variability mediated the relationship between PET-derived measures of dopamine and the brain cognition gap.

Strengths:

The major strength of this manuscript is the extensive effort the authors have put into creating a new 'biomarker' that links deep learning with fMRI, PET, physical activity, education, and cardiovascular risk across two studies. This effort is impressive.

Concerns from the previous round of review:

(1) The primary issue is still the lack of baseline models against which to benchmark the predictive performance of the proposed DenseNet model. This concern was raised independently by two reviewers. Without such benchmarks, it is difficult to interpret the reported results in the context of prior work on MRI-based cognition prediction.

Notably, the authors state: "While we compared our model with the connectome predictive modeling (CPM) approach and observed better performance with our deep learning framework, we did not conduct a comprehensive benchmark across all available machine learning methods, nor was this the aim of the present study."

However, I could NOT find any discussion or results related to the CPM model in the manuscript. It is therefore unclear whether the DenseNet model was actually statistically compared with CPM, and, if so, how the comparison was conducted.

Note that the statement, "While Vieira et al. show that the majority (76%) of prior studies used linear modeling approaches, including CPM and penalized regressions, these models are often vulnerable to overfitting, especially when applied to high-dimensional fMRI data," is not entirely accurate. Linear models typically have far fewer parameters than deep-learning models and are therefore often less prone to overfitting. In fact, it is well established that deep-learning models are particularly susceptible to overfitting and usually require substantially larger sample sizes to achieve stable and reliable performance. Although deep-learning models may outperform shallower models once sufficient data are available and training is well controlled, this does not justify the authors' claim as stated. I therefore disagree with the argument put forward by the authors.

The authors further justify the absence of benchmarking by stating: "In this context, deep learning was employed as a flexible framework capable of modelling high-dimensional functional connectivity patterns across cognitive states, rather than as a claim of inherent methodological superiority. Thus, our goal was not to propose a universally superior prediction model, but rather to test how brain state influences predictive utility for WM and EM using a deep learning approach." However, most shallow models can likewise be applied across different brain states and cognitive targets. This rationale does not establish deep learning as a uniquely appropriate or necessary choice. If deep learning is indeed a better approach in this context, the authors should demonstrate this empirically through appropriate benchmarking against established baseline models.

(2) Additional analysis shows that "BCG is not significantly associated with cognition itself". This is the most perplexing result. This is like saying Brain Age Gap is not related to chronological Age. It is counterintuitive since the Brain Age Gap is calculated by chronological age minus actual age, and most research has shown a strong relationship between the Brain Age Gap and age.

If the brain cognition gap is not related to cognition, is it possible that the results found are mainly due to the predictive model not fitting well with another dataset? Regardless, the lack of association between BCG and cognition deserves a discussion.

(3) I still do not fully understand the rationale of the mediation analysis. The analysis and findings are still not related to aims 1 and 2, since DA and entropy are not part of the prediction models. But I appreciate the explanation that this part is related to the authors' previous work, and that the authors attempted to link to them somehow.

[Editors' note: the authors have responded to these points.]

# Referee report: Reviewer #3 (Public review)

**DOI:** 10.7554/eLife.104053.4.sa3

Summary:

This paper by Esmaeili and co-authors presents a connectome prediction study to predict episodic memory and relate prediction errors to other phonotypic variables.

Strengths:

(1) A primary and external validation dataset.

(2) Novel use of prediction errors (i.e., brain-cognitive gap).

(3) A wide range of data was investigated.

# Author comment: Author response

**DOI:** 10.7554/eLife.104053.4.sa4

**Authors:** Morteza Esmaeili, Erin Beate Bjørkeli, Robin Pedersen, Farshad Falahati, Jarkko Johansson, Kristin Nordin, Nina Karalija, Lars Bäckman, Lars Nyberg, Alireza Salami

**Affiliations:** University of Stavanger Stavanger Norway; Akershus University Hospital Lørenskog Norway; https://ror.org/05kb8h459 Umeå University Umeå Sweden; Karolinska Institutet Stockholm Sweden; https://ror.org/05kb8h459 Umeå University Umeå Sweden; Karolinska Institutet Stockholm Sweden; https://ror.org/05kb8h459 Umeå University Umeå Sweden; Karolinska Institutet Stockholm Sweden; https://ror.org/05kb8h459 Umeå University Umeå Sweden; Karolinska Institutet Stockholm Sweden

The following is the authors’ response to the previous reviews

**Public Reviews:**

**Reviewer #1 (Public review):**

Comments on revised version:

I thank the authors for their extensive efforts to revise the manuscript. I have no further concerns.

**Reviewer #2 (Public review):**

The authors have made several corrections to the original manuscript. For example, they revised the bootstrapping analysis to avoid arbitrarily inflating the degrees of freedom. However, most substantive concerns remain inadequately addressed.

(1) The primary issue is still the lack of baseline models against which to benchmark the predictive performance of the proposed DenseNet model. This concern was raised independently by two reviewers. Without such benchmarks, it is difficult to interpret the reported results in the context of prior work on MRI-based cognition prediction.

Notably, the authors state: "While we compared our model with the connectome predictive modeling (CPM) approach and observed better performance with our deep learning framework, we did not conduct a comprehensive benchmark across all available machine learning methods, nor was this the aim of the present study."

However, I could NOT find any discussion or results related to the CPM model in the manuscript. It is therefore unclear whether the DenseNet model was actually statistically compared with CPM, and, if so, how the comparison was conducted.

Note that the statement, "While Vieira et al. show that the majority (76%) of prior studies used linear modeling approaches, including CPM and penalized regressions, these models are often vulnerable to overfitting, especially when applied to high-dimensional fMRI data," is not entirely accurate. Linear models typically have far fewer parameters than deep-learning models and are therefore often less prone to overfitting. In fact, it is well established that deep-learning models are particularly susceptible to overfitting and usually require substantially larger sample sizes to achieve stable and reliable performance. Although deep-learning models may outperform shallower models once sufficient data are available and training is well controlled, this does not justify the authors' claim as stated. I therefore disagree with the argument put forward by the authors.

The authors further justify the absence of benchmarking by stating: "In this context, deep learning was employed as a flexible framework capable of modelling high-dimensional functional connectivity patterns across cognitive states, rather than as a claim of inherent methodological superiority. Thus, our goal was not to propose a universally superior prediction model, but rather to test how brain state influences predictive utility for WM and EM using a deep learning approach." However, most shallow models can likewise be applied across different brain states and cognitive targets. This rationale does not establish deep learning as a uniquely appropriate or necessary choice. If deep learning is indeed a better approach in this context, the authors should demonstrate this empirically through appropriate benchmarking against established baseline models.

We thank the reviewer for reiterating this point. As noted in both the manuscript and our previous response, the primary goal of the present study was not to benchmark predictive algorithms, but rather to compare the predictive utility of different brain states using a consistent modeling framework.

We did perform an exploratory CPM analysis using a conventional implementation that included correlation-based feature selection (p < 0.01), summarization of positive and negative networks, and robust regression for prediction using 3-fold validation. However, CPM performance can depend substantially on analytical choices, including feature-selection thresholds, treatment of positive and negative networks, cross-validation strategies, and model specification. Although we obtained CPM results (see below), we did not systematically evaluate how these choices influenced performance, nor did we optimize CPM to the same extent as would be required for a rigorous methodological comparison.

For this reason, we chose not to include a CPM-versus-DenseNet comparison in the manuscript. Any direct comparison could easily be overinterpreted as evidence for the superiority of one approach over another, despite the absence of a comprehensive benchmarking framework. We therefore deliberately avoided such claims and instead focused on the scientific question motivating the study: whether predictive performance differs across brain states when the same predictive framework is applied consistently. We agree that comparisons with CPM and other predictive approaches would be valuable, but we believe such analyses are better suited to a dedicated methodological benchmarking study.

(2) Additional analysis shows that "BCG is not significantly associated with cognition itself". This is the most perplexing result. This is like saying Brain Age Gap is not related to chronological Age. It is counterintuitive since the Brain Age Gap is calculated by chronological age minus actual age, and most research has shown a strong relationship between the Brain Age Gap and age.

If the brain cognition gap is not related to cognition, is it possible that the results found are mainly due to the predictive model not fitting well with another dataset? Regardless, the lack of association between BCG and cognition deserves a discussion.

We thank the reviewer for this comment. The absence of a significant BCG-cognition association might be unexpected. We agree it warrants careful interpretation. Theoretically, when a predictive model is trained and evaluated across samples with differing age distributions, the regression-to-the-mean dynamics that typically create BAG–age dependence may not transfer in the same way to BCG–cognition relationships, particularly in age-homogeneous cohorts such as COBRA. However, we acknowledge that the present findings alone are insufficient to resolve this question fully.

We have added additional findings as supplementary material (Figure S4).

(3) I still do not fully understand the rationale of the mediation analysis. The analysis and findings are still not related to aims 1 and 2, since DA and entropy are not part of the prediction models. But I appreciate the explanation that this part is related to the authors' previous work, and that the authors attempted to link to them somehow.

We appreciate the reviewer's comment. The mediation analysis was not intended as a replication of our previous work, but rather as a mechanistic analysis to examine whether the association between BCG and cognition operates indirectly through brain age. Given the established links between BCG, brain age, and cognitive function, mediation analysis provides a principled framework for testing this hypothesis. Essentially, this mediation analysis supports previous theoretical model and our own empirical data where we showed lower DA contributes to more noise in FC metric, which in turn results in less accurate prediction (i.e., larger gap).

**Recommendations for the authors:**

**Reviewer #2 (Recommendations for the authors):**

(1) I hope the authors report CPM analysis as they claimed in the response letter, with actual statistical tests to compare the performance of DenseNet vs. CPM. It will be better to compare DenseNet with other models as well.

We thank the reviewer for reiterating this point. As noted in both the manuscript and our previous response, the goal of the present study was not to benchmark DenseNet against alternative predictive frameworks, but rather to compare the predictive utility of different brain states using a consistent modeling approach. Although we explored CPM as a potential reference model, we found that its performance was sensitive to analytical choices, including feature-selection thresholds, summarization of positive and negative networks, and model specification. A rigorous CPM comparison would therefore require systematic optimization and validation of these choices, which would constitute a separate benchmarking analysis beyond the scope of the present study. We therefore deliberately avoided claims regarding methodological superiority and revised the manuscript accordingly. While comparisons with CPM and other machine-learning approaches would be valuable, we believe such analyses would constitute a separate benchmarking study beyond the scope of the present work.

(2) "n-back did not significantly predict EM in DyNAMiC, and rest did not significantly predict WM. For this reason, we highlighted only the conditions that showed meaningful predictive power in the original analyses."

These null results should also be reported; otherwise, this suggests the authors cherry-picked only the "meaningful" results, making the claims overly optimistic.

We thank the reviewer for this comment. We agree that reporting only significant findings could create the impression of selective reporting. However, this was not our intention. All within-dataset prediction results, including both significant and non-significant findings, are reported in Tables 1 and 2. For the cross-dataset analyses, we chose to evaluate only the best-performing models identified in the DyNAMiC dataset. This decision was made a priori to test whether the most reliable predictive models generalized to an independent dataset, rather than to maximize the number of significant findings. For example, resting-state FC emerged as the strongest predictor of episodic memory in DyNAMiC and was therefore selected for external validation in COBRA. Similarly, the movie-watching model showed the strongest performance for working memory and was consequently carried forward to the cross-dataset validation. We have clarified this rationale in the manuscript.

(3) I appreciate the correlation plots between BCG and physical activity and cardiovascular risk. The results are much weaker in COBRA (r = .17 and -.10 vs. .40 and -.27 in DyNAMIC). Perhaps this warrants discussion. Note that there are potential outliers in DyNAMIC. Perhaps the authors might like to include Spearman's rank.

We thank the reviewer for this helpful observation. We agree that although the associations between BCG and both physical activity and cardiovascular risk were statistically significant in COBRA, the effect sizes were smaller than in DyNAMiC. To assess whether the observed association was influenced by potential outliers, we repeated the analysis using Spearman’s rank correlation. The association remained the same after controlling for age using partial Spearman rank correlation - between GAP and physical activity (DyNAMiC: r = 0.40, p = 0.001; COBRA: r = 0.17, p=0.03) and for GAP vs. CVD risk score (DyNAMiC: r = –0.27, p = 0.03; COBRA: r = –0.10, p = 0.40). Nevertheless, we have now tempered the interpretation in the Discussion (P.12) to clarify that the direction and significance of the associations were consistent across datasets, but that the magnitude of the effects was weaker in COBRA. This difference may reflect cohort differences, including age range, sample composition, and differences in how physical activity was assessed.

(4) Yes, adding figures comparing BCG and BAG in the main text would be helpful, given BAG's popularity.

We have added additional findings as a supplementary figure.

(5) The authors should provide this reason as a justification in the method: "We initially attempted to predict both episodic memory (EM) and working memory (WM). However, EM prediction was only reliable within and across samples for the resting state, whereas WM prediction generalized most strongly from the movie-watching condition. Because COBRA does not include a movie-watching paradigm, we could not evaluate WM prediction across datasets. For this reason, we focused on EM when examining the brain-cognition."

We thank the reviewer’s suggestion. This clarification is included in the method section of the revised manuscript [P 21].
